# Genome-wide genotyping elucidates the geographical diversification and dispersal of the polyploid and clonally propagated yam (*Dioscorea alata*)

**DOI:** 10.1093/aob/mcaa122

**Published:** 2020-06-27

**Authors:** Bilal Muhammad Sharif, Concetta Burgarella, Fabien Cormier, Pierre Mournet, Sandrine Causse, Kien Nguyen Van, Juliane Kaoh, Mamy Tiana Rajaonah, Senanayake Ravinda Lakshan, Jeffrey Waki, Ranjana Bhattacharjee, Gueye Badara, Babil Pachakkil, Gemma Arnau, Hana Chaïr

**Affiliations:** 1 CIRAD, UMR AGAP, F34398-Montpellier, France; 2 AGAP, Univ Montpellier, CIRAD, INRA, Montpellier SupAgro, Montpellier, France; 3 University of Vienna, Department of Evolutionary Anthropology, Vienna, Austria; 4 Uppsala University, Department of Organismal Biology, Uppsala, Sweden; 5 CIRAD, UMR AGAP, Petit Bourg, Guadeloupe, France; 6 Plant Resources Center (PRC), An Khanh, Hoai Duc, Hanoi, Vietnam; 7 Vanuatu Agricultural Research and Technical Centre (VARTC), Espiritu Santo PB, Vanuatu; 8 Kew Madagascar Conservation Centre, Antananarivo, Madagascar; 9 Field Crops Research and Development Institute (FCRDI), Mahailluppallama, Anuradhapura, Sri Lanka; 10 National Agricultural Research Institute (NARI), Lae, Morobe Province, Papua New Guinea; 11 International Institute of Tropical Agriculture (IITA), PMB, Ibadan, Oyo State, Nigeria; 12 Tokyo University of Agriculture (TUA), Sakuragaoka, Setagaya-ku, Tokyo, Japan

**Keywords:** Clonal propagation, demography, geographical distribution, polyploidy, population genomics, yam, *Dioscorea alata*

## Abstract

**Background and Aims:**

Inferring the diffusion history of many human-dispersed species is still not straightforward due to unresolved past human migrations. The centre of diversification and routes of migration of the autopolyploid and clonally propagated greater yam, *Dioscorea alata*, one of the oldest edible tubers, remain unclear. Here, we address yam demographic and dispersal history using a worldwide sample.

**Methods:**

We characterized genome-wide patterns of genetic variation using genotyping by sequencing 643 greater yam accessions spanning four continents. First, we disentangled the polyploid and clonal components of yam diversity using allele frequency distribution and identity by descent approaches. We then addressed yam geographical origin and diffusion history with a model-based coalescent inferential approach.

**Key Results:**

Diploid genotypes were more frequent than triploids and tetraploids worldwide. Genetic diversity was generally low and clonality appeared to be a main factor of diversification. The most likely evolutionary scenario supported an early divergence of mainland Southeast Asian and Pacific gene pools with continuous migration between them. The genetic make-up of triploids and tetraploids suggests that they have originated from these two regions before westward yam migration. The Indian Peninsula gene pool gave origin to the African gene pool, which was later introduced to the Caribbean region.

**Conclusions:**

Our results are congruent with the hypothesis of independent domestication origins of the two main Asian and Pacific gene pools. The low genetic diversity and high clonality observed suggest a strong domestication bottleneck followed by thousands of years of widespread vegetative propagation and polyploidization. Both processes reduced the extent of diversity available for breeding, and this is likely to threaten future adaptation.

## INTRODUCTION

The present geographical distribution of flora and fauna is often the result of a long process of human-mediated dispersal, which had profound effects on the genetic diversity and demographic histories of plant and animal populations (Boivin *et al.*, 2012; Almathena *et al.*, 2016). Inferring the origin of domesticated species and their dispersal is of long-standing interest for evolutionary biologists because it provides information on species’ ability to adapt to environmental changes and opens a window on cultural transitions and spatial expansions in human history. From a practical point of view, addressing the diffusion and diversification of domesticated species also gives insight into diversity that is relevant for breeding programmes and metrics for biodiversity conservation.

After domestication in their centres of origin, most crop species have gone through different waves of translocation through human migrations and therefore have colonized different continents. Archaeological and linguistic data have allowed inference to be made regarding the origin and the dispersal path of several crops (Beaujard, 2011; Fuller *et al.*, 2011; Boivin *et al.*, 2012). Concomitantly, the use of molecular markers has allowed the assumptions made by such studies to be explored through the lens of species genetic make-up and extend the sampling to larger geographical areas (Perrier *et al.*, 2011; Diez *et al.*, 2015). Significant progress in our understanding of crop domestication and evolution has been made in recent years thanks to the development of whole genomic approaches and the availability of genome sequences for many species, such as cassava (Bredeson *et al*., 2016), potato (Hardigan *et al.*, 2017), maize (Kistler *et al.*, 2018) and rice (Choi and Purugganan, 2018).

However, reconstructing the routes of crop translocation is not straightforward due to unresolved human geographical migration and in some cases to the biological specificities of the species itself. For Asian crops, two domestication centres have been identified, one in Southeast Asia such as for rice (Fuller *et al.*, 2009) and common millet (Lu *et al.*, 2009) and one in Papua New Guinea such as for banana (Perrier *et al.*, 2011). These species subsequently spread to different continents through several corridors. The Indian Ocean has been the focus of attention as it is considered one of the most important corridors of wild and domestic plant and animal translocation from Asia to Africa and vice versa, playing an important role in fauna and flora globalization (Boivin *et al*., 2013, 2014). Wallace’s Line, separating the Sunda (Eurasian) and Sahul (mainland Australia, Tasmania and New Guinea) plates, which converged during the late Miocene and Pliocene and resulted in the dispersal of diverse Sunda fauna and flora into the newly formed lowland areas of New Guinea, represents another corridor involved in the still unresolved geographical domestication and translocation of many species between Asia and Oceania (Richardson *et al.*, 2012; Morley, 2018).

Greater yam (*Dioscorea alata*) belongs to the family Dioscoreaceae. Unlike other edible yam species, its pantropical distribution can mainly be explained by its ease of cultivation and broad tolerance to different environments (Orkwor and Asadu, 1998). *Dioscorea alata* is cultivated, for its starchy tubers, in upland parts of Asia (ZhiGang *et al.*, 2014), tropical America (Siqueira *et al.*, 2014), Africa (Egesi *et al.*, 2003; Girma *et al.*, 2014) and in the Pacific (Lebot *et al.*, 1998). In the two latter regions, it is of utmost importance for food security while also being of considerable social and cultural status. In traditional agrosystems, yams are cultivated exclusively through clonal propagation and selection of somaclonal mutants (Vandenbroucke *et al.*, 2016). Flowering is erratic or absent in many cultivars, but new combinations can still be obtained via residual sexual reproduction (Abraham *et al.*, 2013). *Dioscorea alata* is a dioecious, autopolyploid species (2*n* = 2*x* = 40, 3*x* = 60 and 4*x* = 80) (Arnau *et al.*, 2009), and triploids and tetraploids are the result of unreduced gametes (Nemorin *et al.*, 2013). The species is not found in the wild and no wild relatives have been clearly identified (Lebot, 2009). It has been suggested that its closest relative is *D. hamiltonii* based on taxonomic relatedness (Coursey, 1967), or *D. nummularia* based on AFLP markers (Malapa *et al.*, 2005). These hypotheses were later disproved by the studies on chloroplastic markers showing that *D. nummularia* is not the closest relative of *D. alata* (Wilkin *et al.*, 2005; Chaïr *et al.*, 2016). Therefore, its closest relative remains unknown.

The Enantiophyllum clade, which includes *D. alata*, has a Laurasian origin placed in East Asia in the Late Oligocene with *D. alata* speciation dated, using plastid markers, to the late Miocene around 7 Mya (Viruel *et al.*, 2016). The domestication of the species, its introduction to Africa and its worldwide dispersal remain of debate due to the scarcity of genomic data and archaeological remains. The earliest archaeological evidence of the use of greater yam was obtained through an analysis of starch grains extracted from stone artefacts from Papua New Guinea dating back more than 44 000 years (Summerhayes *et al.*, 2010). The greater yam was dispersed from New Guinea by the first Lapita settlers who migrated eastwards, from the Bismarck Archipelago more than 3000 years ago (Kirch, 2000; Bedford *et al.*, 2006). Palaeobotanical scholars linked its introduction in Africa to the joint diffusion of the vegecultural trio ‘taro (*Colocasia esculenta* (L.) Schott), banana (*Musa* sp.) and greater yam’, during the westward expansion of Austronesian-speaking seafarers while colonizing Madagascar through a central Indian Ocean corridor (Fuller *et al.*, 2011). Different *Dioscorea* species are currently found in America including Asian and African species. The introduction of greater yam in America was more likely concomitant with the introduction of other African crops, in the 16th and 17th centuries, following the maritime expansion of Iberians (Carney, 2001).

Few studies have tackled the issue of *D. alata*’s worldwide genetic diversity as most have been conducted at the country level, such as in Brazil (Siqueira *et al.*, 2014), Jamaica (Asemota *et al.*, 1996), China (ZhiGang *et al.*, 2014), Vanuatu (Vandenbroucke *et al.*, 2016; Malapa *et al.*, 2005) and Nigeria (Obidiegwu *et al.*, 2009). The first worldwide study showed a complex pattern of zymotypes shared between continents (Lebot *et al.*, 1998). A recent study analysed a sample from the Caribbean, West Africa, Vanuatu and India with microsatellite markers (Arnau *et al.*, 2017). In Vanuatu yam is known as an important crop and farmers are maintaining its high genetic diversity (Lebot *et al.*, 1998; Vandenbroucke *et al.*, 2016). The highest diversity was encountered in India and Vanuatu, which led to the hypothesis of two centres of diversification (Arnau *et al.*, 2017).

Despite the rising importance of greater yam for food supply, little genomic resources have been developed to understand its domestication and dispersal dynamics. Based on a worldwide sampling spanning four continents and a genome-wide approach, this study aims to explore the demographic history and dispersal of greater yam as well as the contribution of vegetative reproduction and polyploidy to the diversity of this species. Our findings have enabled us to reconstruct the genetic origin and dispersal of greater yam through the Indian and Atlantic oceans.

## MATERIALS AND METHODS

### Sample collection and genotype calling

We collected 600 accessions of greater yam, *Dioscorea alata* L., from the species main growing areas in Asia: India, Japan, Sri Lanka and Vietnam (222); Africa: Benin, Burkina Faso, Congo, Côte d’Ivoire, Equatorial Guinea, Ghana, Madagascar, Nigeria, Sierra Leone and Togo (141); the Caribbean: Cuba, Dominican Republic, Guadeloupe, Haiti, Jamaica, Martinique, Puerto Rico, Saint Lucia and Saint Vincent (157); and the Pacific: New Caledonia, Papua New Guinea and Vanuatu (123) (Supplementary Data Fig. S1). As we had just three accessions and one accession from French Guyana and Brazil, respectively, we pooled them with the Caribbean accessions to simplify the analysis (Fig. S1). To set a threshold for clonal lineage identification, the sampling included two progenies of 15 accessions each with one shared parent, that were previously used to build up the *D. alata* genetic map (Cormier *et al.*, 2019) along with an additional 25 known hybrids and replicates. DNA extraction and genotyping by sequencing (GBS) were performed as described by Cormier *et al.* (2019). GBS libraries were constructed as described by Elshire *et al.* (2011) using *Pst*I-*Mse*I restriction enzymes (New England Biolabs, Hitchin, UK) on 200 ng of genomic DNA for eacch sample, followed by ligation with a barcode adapter and a common Illumina sequencing adapter. Amplified multiplexed libraries were purified and verified to ensure that most of the DNA fragments were between 150 and 300 bp. Sequencing was conducted on an Illumina HiSeq 3000 system (150-bp, single-end reads) at the GeT-PlaGe platform in Toulouse, France.

Fastq files were demultiplexed with GBSX, and Illumina adaptators were then removed from the sequences using Cutadapt v1.9 (Martin, 2011). A total of 5 925 921 969 raw reads were obtained from the 643 accessions, available in the NCBI SRA (Sequence Read Archive), under BioProject number PRJNA576311. Single nucleotide polymorphism (SNP) calling was performed using the VcfHunter package (Garsmeur *et al.*, 2018) consisting of a mapping step on the *D. rotundata* reference genome (Tamiru *et al*., 2017) using the ‘process_reseq’ pipeline, followed by site pre-filtering using the VcfPreFilter.1.0.py program with default parameters. SNP calling was done separately for each chromosome and 21 variant call format (VCF) files were generated and then concatenated to produce a single final VCF file.

SNPs were filtered using VCFtools v0.1.14 (Danecek *et al.*, 2011) based on the following criteria: minimum read depth 8, minimum allele count 3, minimum allele frequency 0.05, biallelic loci only and maximum missing rate of 10%, leading to a genotyping matrix of 643 genotypes and 6017 SNPs. To build the site frequency spectrum (SFS) for demographic analysis, the same parameters were used on 72 non-clonally related diploid genotypes from Asia, Africa and the Pacific, identified subsequently without applying the minimum allele frequency filtering, leading to 384 469 SNPs.

### Ploidy-level inference from the GBS data

The ploidy level of the accessions received from the different sources was mostly unknown. It was thus crucial to first assess it. We designed a method based on the allelic frequency distribution at heterozygotic loci (PolynomPloidy.R) (Supplementary Data Method S1). First, the allelic frequency per accession and per site was calculated by dividing the number of allele reads by the total number of reads. The distribution of these observed allelic frequencies was then plotted as a barplot (Fig. S2) on which a polynomial function was fitted with no prior on its mode (e.g. unimodal, bimodal or trimodal). Finally, the ploidy level was inferred from the number of local maxima of the fitted polynomial function. Indeed, for a diploid individual, a normal distribution centred at mean = 0.5 was expected, whereas for a triploid or tetraploid the expected distributions were bimodal (⅓, ⅔) and trimodal (¼, ½ and ¾), respectively. Accessions for which the fitted polynomial function did not contain any local maximum or more than three were set as unknown ploidy.

Different parameters/thresholds (e.g. number of reads per site, minor allele frequency) were tested to filter the GBS data and barplots were drawn using 100, 250 or 500 classes (Supplementary Data Table S1). All combinations of parameter values, thresholds and barplot classes were tested and the combination that maximized the accuracy on a training dataset was then used on the whole dataset. Indeed, a training phase was first conducted using 33 accessions consisting of 15 diploids, eight triploids and ten tetraploids, as determined by flow cytometry on which the combinations of parameters values that maximized accuracy well predicted 78% of ploidy levels.

### Relatedness between diploid and polyploids

The genotyping matrix (643 genotypes × 6017 SNPs) was converted into a 012 incidence matrix with 0, 1 and 2 corresponding to homozygote for the reference allele, and heterozygote and homozygote for the alternate allele, respectively. Three matrices were produced, the first with the whole dataset, the second after removal of accessions with unknown ploidy (487 genotypes), and the third including the 93 independent diploid accessions (i.e. after removing multi-locus genotypes, see below) as well as the triploid and tetraploid accessions. Missing data were imputed using the mean allele frequencies. Kinship between samples was calculated as 1 minus the pairwise genetic distance derived from the Manhattan function and thus assessed as a percentage of common alleles. Significant kinship between samples was detected fitting a normal law on the kinship distribution between independent diploids (*N* = 93) using the fitdist function of the fitdistrplus R package (Delignette-Muller and Dutang, 2015). The significance threshold was set at a *P* = 0.05. These analyses were conducted under the R CRAN 3.4.0 environment (R Core Team). Finally, a network visualization based on significant kinship was drawn up using the prefuse force directed layout implemented in the Cytoscape 3.4.0 software (Shannon *et al.*, 2003).

### Duplicate identification

To identify multi-locus lineages (MLLs) represented by distinct multi-locus genotypes (MLGs), kinship coefficient, π, and identity-by-descent (IBD) probabilities for all possible pairs among the 347 diploid accessions were calculated using PLINK software (Purcell *et al.*, 2007), as described for grape (Myles *et al.*, 2011). Information from the two offspring cohorts (full and half siblings) was used to set the threshold for clones, i.e. the valley between the non-related accessions and the closely related accessions, probably parent–offspring (Fig. S3A), and the non-related accessions but genetically close as result of clonal multiplication (Fig. S3B). After removing progenies, hybrids and replicates, an index of genotypic richness (*R*) was calculated as described by Dorken and Eckert (2001) along with the fixation index *Fis*. We then generated a sample with unique diploid genotypes and randomly selected one MLG representative from each MLL.

### Genetic population analyses

The 93 unique diploid accessions were used to investigate population structure. Diversity statistics – nucleotide diversity, Tajima’s *D* (Tajima, 1989) and *Fst* (Weir and Cockerham, 1984) – were calculated and averaged on 100-kb genomic bins containing at least three SNPs using VCFtools v0.1.14 (Danecek *et al.*, 2011). Kruskal–Wallis rank sum and pairwise Wilcoxon tests were performed to assess their significance. Principal component analysis (PCA) was carried out using the *FactoMiner R* package (Lê *et al.*, 2008). Population structure was calculated using ADMIXTURE (Alexander *et al.*, 2009), testing replicates of five at *K* = 2 to 6. After selection of the optimal number of *K* reflecting the most probable number of groups, an 80% ancestry threshold was set to assign each individual genome to a group. Accessions with membership probabilities under 80% were considered to be of admixed origin.

### Demography modelling

To more thoroughly investigate the genetic history of the identified gene pools and their evolutionary relationships, coalescent simulations were performed to compare different demographic scenarios using FASTSIMCOAL v2.6 (Excoffier *et al.*, 2013). To build the SFS, 384 469 SNPs were used after filtering for quality parameters without removing the rare variants. To account for missing data, we estimated the allele frequency by downsizing the sample size of each population to the value of the smallest population in our data set, and we built 2-D SFSs, adapting the customized R-script used by Burgarella *et al.* (2018). The likelihood of each model and the parameter estimations were optimized using 100 independent runs, each using 1 million simulations and 100 cycles of the conditional maximization algorithm (ECM). For each model, we used the point estimate of parameters resulting in the best likelihood value from the best run of the first round of simulations to refine the likelihood estimation. Likelihoods were re-estimated for each model using 100 additional runs of 1 million simulations. Then, Akaike’s Information Criterion (AIC) was calculated for model comparison.

First, three simple demographic models were tested, featuring Mainland Southeast Asia (MSEA), Indian Peninsula and Pacific gene pools. Migration was then added between the Indian Peninsula and the Pacific in the two best topologies obtained from previous analyses. After analysing the relationship between these three populations, Africa was included in the best model obtained previously and three further models were tested. The Caribbean sample was not included in this analysis as yam was introduced to the Americas relatively recently, primarily from Africa via the slave trade (Carney, 2001). Each model is described in Supplementary Data Method S2.

## RESULTS

### Distribution of ploidy levels across continents

GBS of all samples produced more than 5 billion raw reads that were mapped to the *D. rotundata* V1 reference genome (Tamiru *et al.*, 2017), resulting in the identification of 15 048 820 SNPs. Then, filters of different stringency were applied depending on the requirements of each performed analysis (Material and Methods).

To define individual ploidy level and sample clonal structure, we used 6017 high-quality bi-allelic SNPs well distributed across the 20 *D. alata* linkage groups (Cormier *et al.*, 2019) (Supplementary Data Fig. S4). We computed the distribution of allele frequencies per accession at heterozygous loci, and were able to infer the ploidy of 487 (75.7%) accessions. We determined that 352, 100 and 34 accessions were diploid, triploid and tetraploid, respectively (Table 1). Of the 352 diploids, 302 accessions were landraces and 50 were replicates and hybrids (Table S2). No significant difference was observed in the distribution of diploid accessions among continents. In contrast, the number of triploids in Asia and the Caribbean was higher than in Africa and the Pacific. For 162 accessions (25.19 %), the model was unable to define the ploidy level due to the sequencing depth. Indeed, a stringent threshold of minimum depth per accession and per site (30×) was defined as an optimal parameter during the model training (Table S1).

**Table 1.** The number of accessions per continent according to the ploidy level inferred from GBS data and the genetic parameters for diploid landraces.

Continent		Ploidy in the whole dataset (%)				Genetic parameters for diploid landraces					
	*N*_t_	2*x*	3*x*	4*x*	NA	*N*_2*x*_	UG	MLL	G	*R*	*Fis*
Africa	141	44.68	9.92	7.09	38.29	63	6	13(7)	19	17.98	−0.16
Asia	222	57.20	18.47	3.15	21.17	118	20	21(4)	41	39.99	−0.15
Caribbean	157	66.87	15.92	3.18	14.01	65	18	13(12)	31	29.98	−0.07
Pacific	123	46.34	13.00	9.75	30.89	56	9	12(8)	21	19.98	−0.14
Total	643	54.74	14.93	5.28	25.03	302	53	–	112	–	–

*N*_t_, sample size; *N*_2*x*_, sample size for diploids (after removing full and half-siblings, and replicates); UG, number of unique genotypes; MLL, number of multi-locus lineages, with shared MLLs with another continent in parentheses; G, number of independent genotypes; *R*, genotypic richness index; *Fis*, fixation index per continent.

**Fig. 1.**
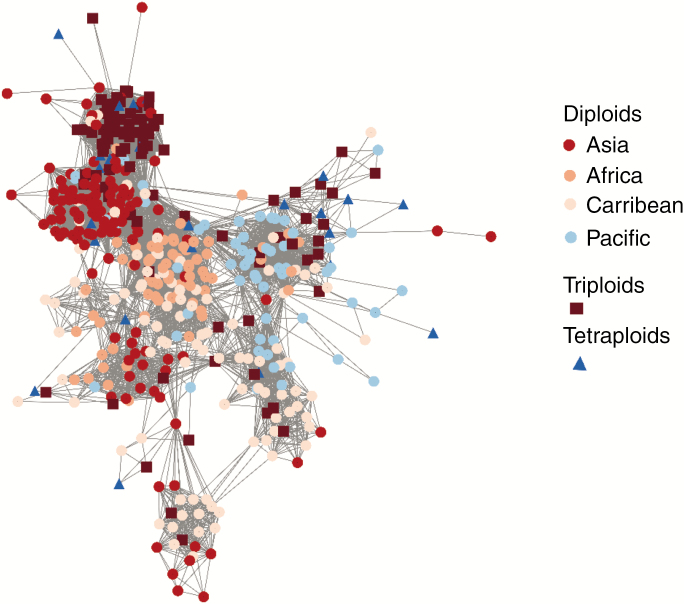
Network showing the genetic relationships between diploid, triploid and tetraploid accessions. The combination of colours and shape represents ploidy levels and geographical origin.

The genetic distance-based network computed to assess the geographical and genetic origin of polyploids showed that those accessions with unfitted ploidy level were spread evenly over the network, in agreement with the lower depth of sequencing rather than the geographical or phylogenetic relatedness and were thus removed (Supplementary Data Fig. S5). Diploids belonging to the same geographical origin were clustered (Fig. 1). Most triploid accessions clustered in two groups. One was close to diploids from Asia (mostly accessions from Vietnam). Their genetic similarity suggests that there were very few clonal lineages. This group included 60 of the 100 triploid accessions from Asia (34 out of 41), Africa (six out of 14) and the Caribbean (17 out of 29). The second triploid group (17 out of 100) was close to accessions from the Pacific, with more genetically distant genotypes, suggesting a higher rate of different polyploidization events. The tetraploid genotypes were close to these two main triploid groups. The genetic proximity between diploids, triploids and tetraploids from the same geographical origin suggested that the polyploids were derived from unreduced gametes from the same diploid gene pool. The few remaining triploid and tetraploid accessions were close to the African and Caribbean diploid gene pools, which may be explained by tuber dispersal via human migrations.

Populations of mixed ploidy cannot be assessed using current statistical approaches (Meirmans *et al.*, 2018). As reported in previous studies and confirmed here, the natural frequency of diploid lineages outweighs the contribution of polyploids to diversity (Abraham *et al.*, 2013). Moreover, genetic diversity within the diploid gene pool well represented the overall diversity of the species as polyploids arise from diploids through unreduced gametes formation (Nemorin *et al.*, 2013). We thus focused the subsequent analyses on the 347 diploid accessions.

### Clonal relationship between diploid accessions

**Fig. 2.**
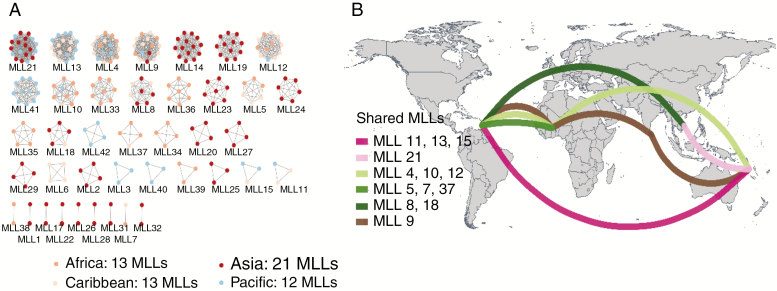
(A) Clonal relationships within the 40 multi-locus lineages (MLLs) identified among the 302 *Dioscorea alata* diploid accessions. Each cluster represents one MLL. Node colours correspond to the geographical origin of the clones. –(B) Geographical distribution of MLLs shared between continents. The total number of MLLs within each continent is reported: Africa 13 (shared seven); Asia (shared four); Caribbean 13 (shared 12) and Pacific 12 (shared eight). (See also Table 1.)

After calculating the IBD density distribution of all possible pairs of the 352 diploid accessions and setting the clonality threshold, only 53 landraces were identified as unique genotypes (UGs). The other 249 landraces had at least one clone and were spread over 40 MLLs, containing between two and 19 accessions (Fig. 2A). The 302 diploid landraces were thus placed into only 93 independent diploid genotypes (40 MLLs and 53 UGs), which highlighted high clonality within the species. The high extent of clonality was confirmed by the low genotypic richness (*R* = 17.98–39.99) and by the negative inbreeding coefficients (excess of heterozygosity) within continents (Table 1).

Among the 40 MLLs, only one MLL had clones shared between the four continents while 13 had an intercontinental distribution (Fig. 2B). Most of the MLLs (68%) had an intracontinental distribution with 19 exclusive to a single country. The Caribbean had the highest number of MLLs shared across continents (12/13), while Asia had the lowest (4/13). The non-homogeneous geographical span of some of the clonal lineages could be the consequence of their adaptation to a particular environment vs. a wide range of environments, although sampling bias cannot be excluded.

### Population structure and nucleotide diversity

**Fig. 3.**
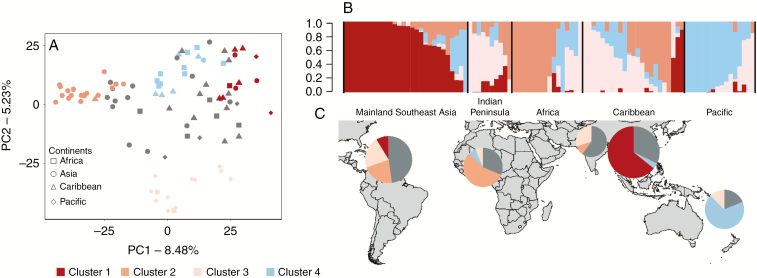
Visualization of genetic relationships between the 93 diploid *Dioscorea alata* accessions from Africa, Asia, Caribbean and Pacific after removing the clones. (A) Principal component analysis depicting the 93 accessions. Squares, Africa; circles; Asia; triangles, Caribbean; and diamonds, Pacific. Accessions are coloured according to their assignment to the four genetic clusters after Admixture analysis. The threshold to assign a genotype to a cluster is set at 80 %. (B) Admixture barplot showing the distribution of the *K* = 4 genetic clusters. Within each continent, accessions are ordered according to cluster assignment proportions. (C) Map showing the geographical distribution of the 93 accessions in each continent according to their genetic clustering.

Within each MLL, a single genotype representative of the clonal lineage was randomly chosen and added to the 53 unique genotypes, leading to a set of 93 diploid genotypes that were used for the following analyses. We first assessed the population structure through PCA. PC1 separated the Asian accessions from the rest while PC2 distinguished the Pacific samples (8.48 and 5.23 % of the variance explained, respectively) (Fig. 3A). This genetic structure was confirmed by the ADMIXTURE analysis (Fig. 3B). We set an 80% ancestry threshold for the assignment of individuals to a genetic group, which resulted in four clusters (Supplementary Data Fig. S6). In the first cluster, 90 % of the accessions were from Mainland Southeast Asia (mainly Vietnam). The second cluster included two accessions from India and Sri Lanka, four from the Caribbean and one from Africa and the Pacific. The third cluster was mainly from the Pacific region (77 %) and the fourth cluster was mainly from Africa (66.6 %) and the Caribbean (25 %). The remaining 42 accessions were of admixed ancestry between the three clusters, suggesting a complex history among these geographical areas. Because there was a good correspondence between genetic structure and geographical origin, and we sought to determine the origin and dispersal of yam among continents, we subdivided our sample in five gene pools for the subsequent analyses: Mainland Southeast Asia (MSEA), Indian Peninsula (InP), Pacific (Pac), African (Afr) and Caribbean gene pools (Supplementary Data Table S3). Nucleotide diversity was low in all gene pools, with the highest values obtained for the Pacific and MSEA (π = 1.29e^−5^ and 1.26e^−5^ respectively) and the lowest for the Caribbean and Africa (π = 0.96e^−5^ and 1.10e^−5^, respectively) (Table 2). A negative Tajima’s *D* was obtained for all gene pools, indicating an excess of low-frequency alleles, relative to intermediate frequencies, a signal that could reflect demographic expansion as well as clonal reproduction. The highest negative values were obtained for Caribbean and Indian Peninsula gene pools (*D* = −0.57 and −0.43, respectively) with lower values obtained for MSEA and the Pacific (*D* = −0.07 and −0.12, respectively. The *Fst* values were very low (*Fst* = 0.001–0.055), indicating a weak but generally significant differentiation between the gene pools (Table 2). The lowest *Fst* value was obtained between Africa and the Caribbean and the highest between the MSEA and Indian Peninsula gene pools.

**Table 2.** Population genetic statistics for the 93 diploid genotypes per geographical origin.

Continent	*π* (IQR)	*D* (IQR)	*Fst* Africa	*Fst* MSEA	*Fst* Indian Peninsula	*Fst* Caribbean
All	0.84e^−5^ (0.92e^−5^)	−1.06 (0.98)	–	–	–	–
Africa	1.10e^−5^ (0.87e^−5^)	−0.14 (1.4)	–	–	–	–
MSEA	1.26e^−5^ (0.97e^−5^)	−0.08 (1.35)	0.03	–	–	–
Indian Peninsula	1.20e^−5^ (0.94e^−5^)	−0.43 (1.14)	0.026	0.055	–	–
Caribbean	0.96e^−5^ (0.81e^−5^)	−0.57 (1.19)	0.001	0.023	0.011	–
Pacific	1.29e^−5^ (0.97e^−5^)	−0.12 (1.4)	0.019	0.037	0.03	0.015

π, Nucleotide diversity; *D*, Tajima’s *D*; IQR, interquartile range.

### Inference of demographic history

**Fig. 4.**
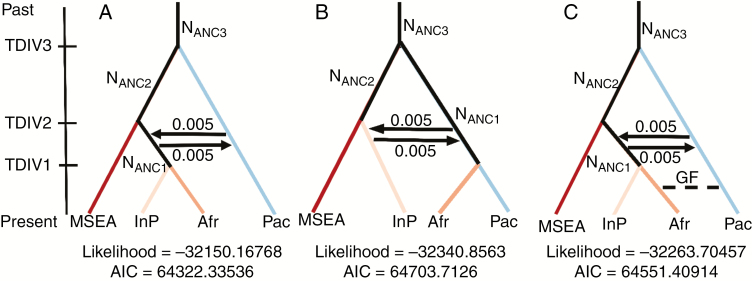
Demographic scenarios of domestication simulated with FASTSIMCOAL2.6. The split between Mainland South East Asia (MSEA) and the Pacific (Pac) was followed by the split of Indian Peninsula (InP) populations with constant migration between InP and Pac. Scenarios: (A) origin of Africa (Afr) from the Indian Peninsula, (B) origin of Africa from the Pacific, (C) origin of Africa from the Indian Peninsula with gene flow between the Pacific and Africa. N_ANC_, ancestral population size. TDIV, time of divergence; TDI1 < TIV2 < TDIV3. GF, gene flow. 0.005 is the migration rate between two populations.

First, we assessed the most likely tree topology for the MSEA, Indian Peninsula and Pacific gene pools in the presence and absence of gene flow. First, three simple demographic models were tested, featuring the MSEA, Indian Peninsula and Pacific gene pools. Migration was added between the Indian Peninsula and the Pacific in the two best topologies obtained from previous analyses. After analysing the relationship between these three populations, an AIC-based model comparison supported a scenario in which the Pacific lineages split early from an Asian ancestral population. Asian lineages split later into MSEA and IndP, and continuous migration occurred between Indian and Pacific gene pools (Supplementary Data Fig. S7a–e). We then used this inferred scenario to determine the origin of the African gene pool by comparing three scenarios whereby Africa originated from Pacific or IndP or both (Fig. 4). We did not test an African origin from MSEA because previous studies provided evidence of the colonization of Madagascar and Africa from Southeast Asia, mainly through the Austronesians (Boivin *et al.*, 2013; Crowther *et al.*, 2016) and our ADMIXTURE analysis did not support common ancestry between the MSEA and African accessions. Indeed, no African accessions were assigned to the MSEA gene pool and only one was assigned to the Pacific one (Fig. 3; Table S3). This analysis yielded evidence of the introduction of the African gene pool from the Indian one, and excluded major contributions from the Pacific gene pool (Fig. 4A).

## DISCUSSION

Investigating the evolutionary history of a crop such as *Dioscorea alata* is a challenging task for multiple reasons. Greater yam is unknown in the wild and its wild relatives have yet to be identified. The origin of greater yam domestication and its dispersal are closely tied to three main historical events that are still under investigation, i.e. the Sunda or Sahul (mainland Australia, Tasmania and New Guinea) domestication origin of many crops, human migrations from Asia to Africa via the Indian Ocean and the more recent Colombian exchange. We advanced our understanding of *D. alata* diversity and evolution by analysing the widest and most comprehensive sample of greater yam to date, spanning the four main continents where the species is cultivated.

### Clonality as factor of yam diversification

We showed that worldwide diversity of greater yam is characterized by a high extent of clonality, corroborating findings from our previous studies (Arnau *et al.*, 2017). Indeed, the 302 diploid accessions in our sampling were actually derived from only 93 independent genotypes (70 %), which is in line with the fact that greater yam has long been cultivated by vegetative propagation in farming systems so diversification has mainly occurred by somaclonal mutation. Our results were therefore the direct consequences of this somaclonal selection and allowed us to quantify it. Farmers collect tubers in fallow, evaluate and then select them during cultivation by vegetative propagation. This is a common practice in root and tuber crop farming (McKey *et al.*, 2010), and has been described in African yam (Chaïr *et al.*, 2010) and cassava (Duputié *et al.*, 2009).

### Genetic and geographical origin of yam polyploids

We found that the most common forms were diploids, followed by triploids and tetraploids, in agreement with previous studies (Abraham and Nair, 1991; Arnau *et al.*, 2009). Indeed, triploid genotypes are created from a rare single unreduced gamete, while two unreduced gametes are required for tetraploid formation, which is even less frequent (Nemorin *et al.*, 2013). The reduced number of triploids and tetraploids relative to diploids could thus be explained by the rare occurrence of autopolyploidization added to the erratic flowering of diploids. As most polyploid accessions were genetically close to diploids from the Asian or Pacific gene pools, independently of their geographical origin, we could reasonably hypothesize that polyploidization occurred several times independently in these two regions before the migration of greater yam from Asia and the Pacific to Africa and the Caribbean. Interestingly, most of the African and Caribbean triploid accessions appeared to be close to diploid Asian accessions, suggesting that the Asian gene pool contributed more than the Pacific one to westward diffusion of greater yam. The high genetic similarity found within the Asian triploid accessions suggested that they arose from a few autopolyploidization events, which have been multiplied by vegetative propagation. In the Pacific region, where yam is an important staple food crop, unlike in Asia where rice is predominant, the diversity observed in polyploids might be the result of preservation of greater diversity by farmers, or recent polyploidization events such as for cassava in Vanuatu where recruitment of spontaneous triploid accessions by farmers is still ongoing (Sardos *et al.*, 2009).

### Genetic evidence of yam early diffusion

*Dioscorea alata* harboured very low nucleotide diversity in comparison to potato diploid landraces (Hardigan *et al.*, 2017) or cultivated and wild cassava (Ramu *et al.*, 2017). It is also generally recognized that the reduction in sexual fitness is a domestication trait in asexually propagated crops (Meyer and Purugganan, 2013; Denham *et al.*, 2020), as recently shown in potato (Hardigan *et al.*, 2017). Consequently, despite the absence of identified wild relatives, based on the narrow nucleotide diversity, the high number of clonal lineages and the scarcity of flowering observed in the field, we could reasonably deduce that greater yam has undergone a strong domestication bottleneck. Most previous studies have focused on the Indian and Pacific regions, which generated incomplete and contrasting pictures of the domestication origin of *D. alata*. By expanding the sampling beyond these areas, we revealed an early split between the Mainland Southeast Asia and Pacific gene pools and later the split between MSEA and Indian Peninsula. Our demographic inference findings indicated continuous migration between the Pacific and Indian Peninsula regions, which might also explain the divergence in previous hypotheses on the origin of *D. alata* (Arnau *et al.*, 2017). We could not date the split between the MSEA and Pacific gene pools. However, considering that the peopling of Sahul dates back to at least 50 000 years ago (Bird *et al.*, 2019), and that hunter-gatherer societies exploited endemic yams in New Guinea 49 000–36 000 years ago (Summerhayes *et al.*, 2010), it is likely that yam had reached the Sahul in wild or pre-domesticated form and was domesticated later in both the MSEA and the Pacific regions. Similarly, multiple geographical domestication regions have been suggested for different crops such as maize (Kistler *et al.*, 2018) and barley (Dai *et al.*, 2014). Greater yam would subsequently have been dispersed eastwards by the first Lapita settlers (Kirch, 2000; Bedford *et al.*, 2006).

### African gene pool originating from the Indian Peninsula

Demographic analysis indicated an Indian Peninsula origin of the African yams. None of the African accessions was assigned to the MSEA genetic pool and only one was assigned to Pacific one. The same applies to accessions from Madagascar, which is assumed to be one of the gates by which yam reached Africa (Beaujard, 2011), as all but one were assigned to the Indian Peninsula gene pool. Moreover, only two polyploids were found to be genetically close to the Pacific gene pool while all African triploid and tetraploid accessions were close to the Asian gene pool. This is in accordance with an introduction of yam in Africa via the Indian Ocean. African accessions had the lowest levels of nucleotide diversity, suggesting a strong founder effect. While we obtained genetic evidence of the Indian Peninsula origin of greater yam in Africa, the routes of its introduction could not be traced from this study. Two main routes are often discussed, one involving an introduction through Madagascar followed by the colonization of Africa, and a second assuming an entry from East Africa, mainly via the Swahili coast (Boivin *et al.*, 2013). The importance of greater yam cultivation in Madagascar is well documented, even though this crop has gradually been substituted by rice (Beaujard, 2011; Crowther *et al.*, 2016). How yam, banana and taro all reached West Africa remains unclear but probably occurred much later than the first introductions in East Africa.

### From Africa to the Carribean: the slave trade as a means of yam introduction to the Americas

We gathered different lines of evidence supporting that the Caribbean gene pool mainly originated from Africa. Genetic differentiation was almost null between African and the Caribbean diploid gene pools. Most of the accessions were assigned to the Indian Peninsula and African gene pools. Moreover, most of the clonal lineages were shared with Africa. Greater yam was most probably introduced to the Americas during the Colombian exchange. The slave trade is known to have been a factor of introduction of African crops to the tropical Americas during Colombian exchange (Boivin *et al.*, 2012). In fact, yams had provisioned the slave ships that traversed the Middle Passage of Atlantic slavery for some 350 years (Carney, 2001). Reports have indicated the introduction of African yams but without providing any taxonomic information on the species involved. The greater yam could have been introduced with Guinea yam (*D. rotundata*). While African rice (*Oryza glaberrima*), probably introduced concomitantly with yams, has now been supplanted by Asian rice (*O. sativa*) (van Andel *et al.*, 2016), greater yam remains the most widely cultivated species in the tropical Americas.

## CONCLUSIONS

In the present study, our genetic analysis and demographic inference supported an early divergence of greater yam between Mainland Southeast Asia and the Pacific, probably followed by two independent domestication events. The species would then have reached the Indian Peninsula, subsequently Africa and from there the Caribbean. We also revealed high clonality and low nucleotide diversity, which are indicators of a strong domestication bottleneck and a diversification process achieved mainly via somaclonal accumulation. The narrow diversity raises concerns about the scope for genetic improvement of traits of interest. Future research efforts should have the two-fold aim of exploring the adaptation of worldwide-distributed clonal lineages under different cropping systems and environmental constraints, and identify useful alleles within the untapped diversity of greater yam wild relatives.

## SUPPLEMENTARY DATA

Supplementary data are available online at https://academic.oup.com/aob and consist of the following.Fig. S1: Geographical origin of the 643 accessions of *Dioscorea alata*. Fig. S2: Bar plot of the allelic frequency distribution and the corresponding number of chromosomes after chromosome counting for diploid, triploid and tetraploid accessions. Fig. S3: The density distribution of identity by descent for all possible pairs of accessions, full-sibs and half-sibs, and the frequency distribution of all possible pairs of accessions with a defined window. Fig. S4: Distribution of the 6017 SNPs along the *Dioscorea alata* genetic map. Fig. S5: Network visualization of the genetic relationships between the 643 accessions according to their ploidy level and their geographical origin. Fig. S6: Cluster assignment of 93 diploid greater yam genotypes estimated using ADMIXTURE. Fig. S7: The different scenarios of domestication simulated with FASTSIMCOAL2.6. Table S1: Description of the optimal set of parameters used on the whole dataset and defined in the model training for inferring the ploidy level from GBS data. Table S2: *Dioscorea alata* accessions used in this study. Table S3: Geographical distribution of diploid genotypes showing estimated proportion of membership of their genome in each of the four gene pools. Method S1: PolynomPloidy.R script. Method S2: Description of the demographic models tested.

mcaa122_suppl_Supplementary_Material
