# Supplementary material for: Genome-wide genotyping elucidates the geographical diversification and dispersal of the polyploid and clonally propagated yam (Dioscorea alata)
Source: Ann Bot. 2020 Jun 27;126(6):1029–38. doi: 10.1093/aob/mcaa122 (PMC7596366; doi:10.1093/aob/mcaa122)
Supplement: mcaa122_suppl_Supplementary_Material [file mcaa122_suppl_supplementary_material.docx]

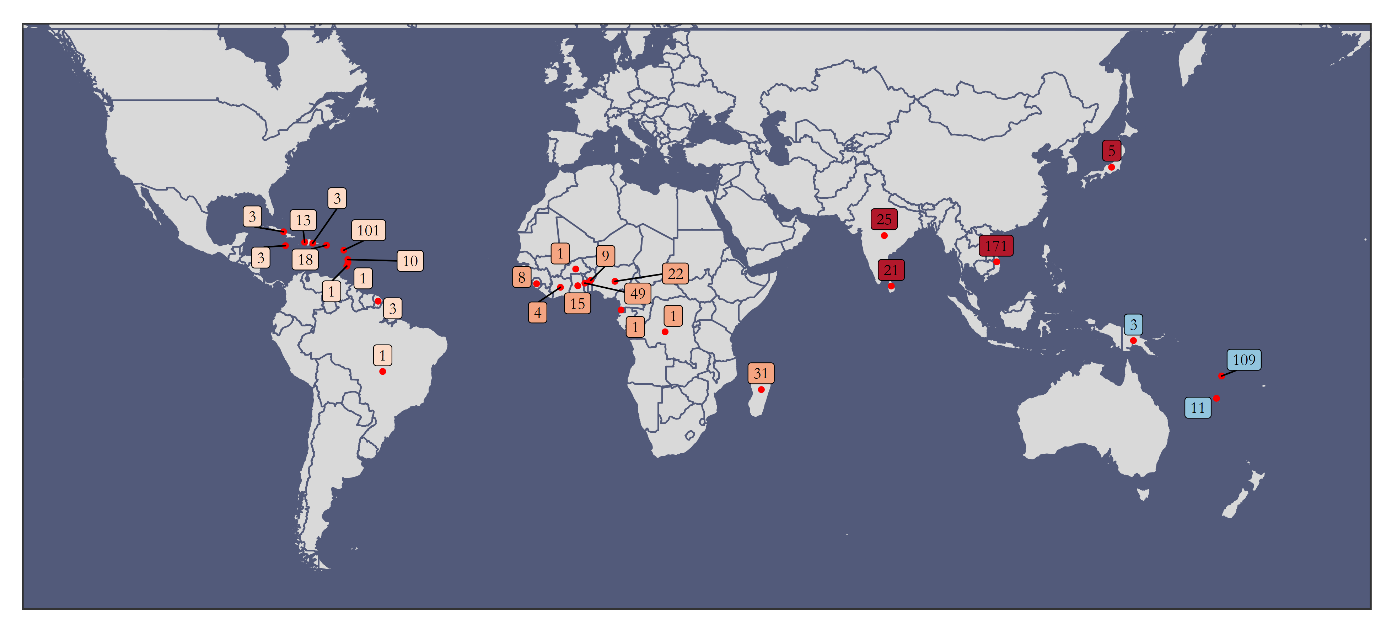


**Fig. S1** : Geographical origin of the 643 *Dioscorea alata* accessions.


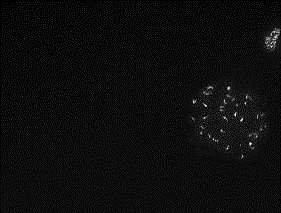

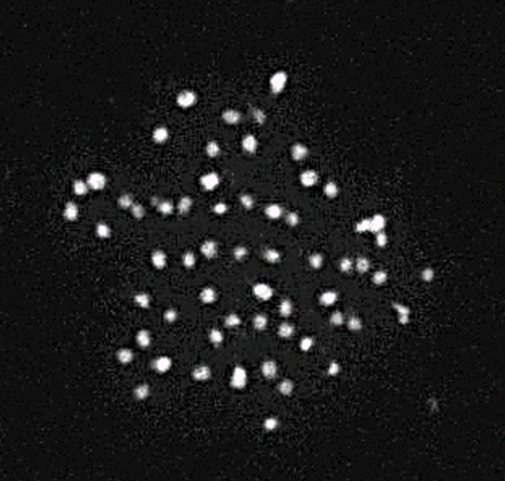

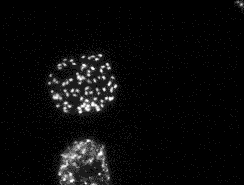

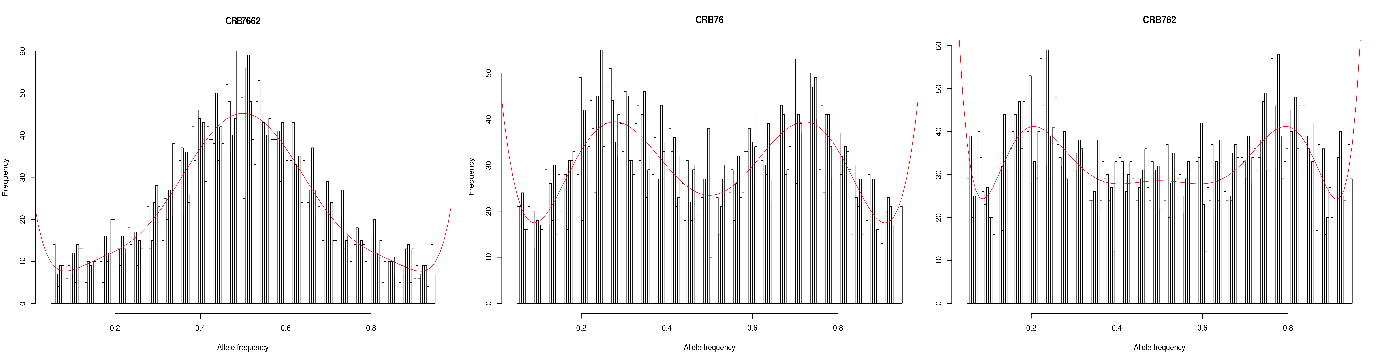


**Fig. S2**: Bar plot of the allelic frequency distribution and the corresponding number of chromosomes after chromosome counting for diploid, triploid and tetraploid accessions, from left to right, respectively.

**Table S1:** Description of the optimal set of parameters used on the whole dataset and defined in the model training for inferring the ploidy level from GBS data.

| **Parameters** | **Min** | **Max** | **Step** | **Optimal** |
| --- | --- | --- | --- | --- |
| Maximum number of reads per site | 75000 | 150000 | 25000 | 150000 |
| Minimum number of reads per site and per accession | 10 | 50 | 10 | 30 |
| Maximum number of reads per site and per accession | 200 | 800 | 200 | 800 |
| Maximum allelic frequency of major alleles | 0.85 | 1 | 0.05 | 0.95 |

**A B**

| 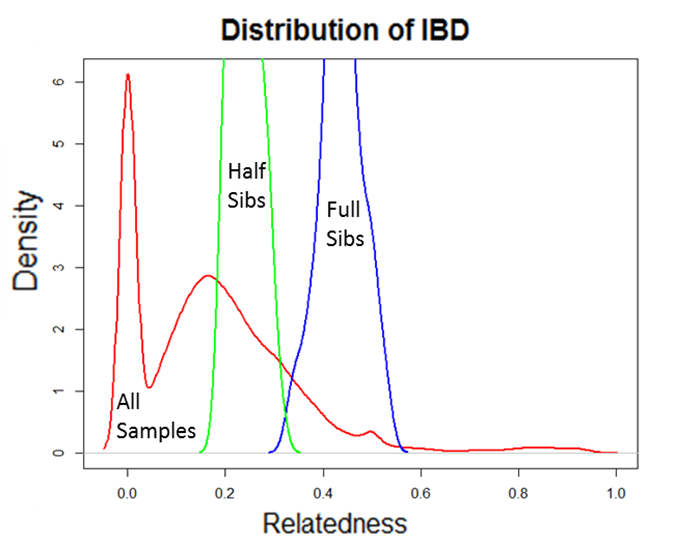 | 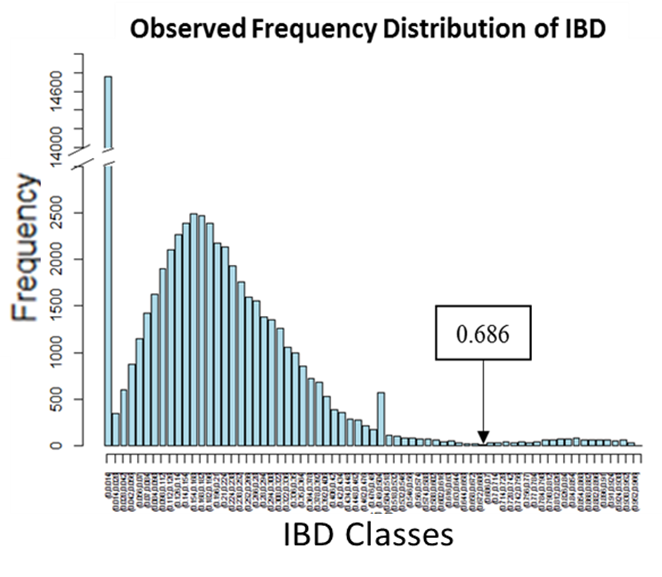 |
| --- | --- |

**Fig. S3** (A) The identity by descent (IBD) density distribution for all possible pairs of accessions, full-sibs and half-sibs; (B) The frequency distribution of all possible pairs of accessions with a defined window of 0.014 and a valley at 0.686 indicating the clonality threshold.


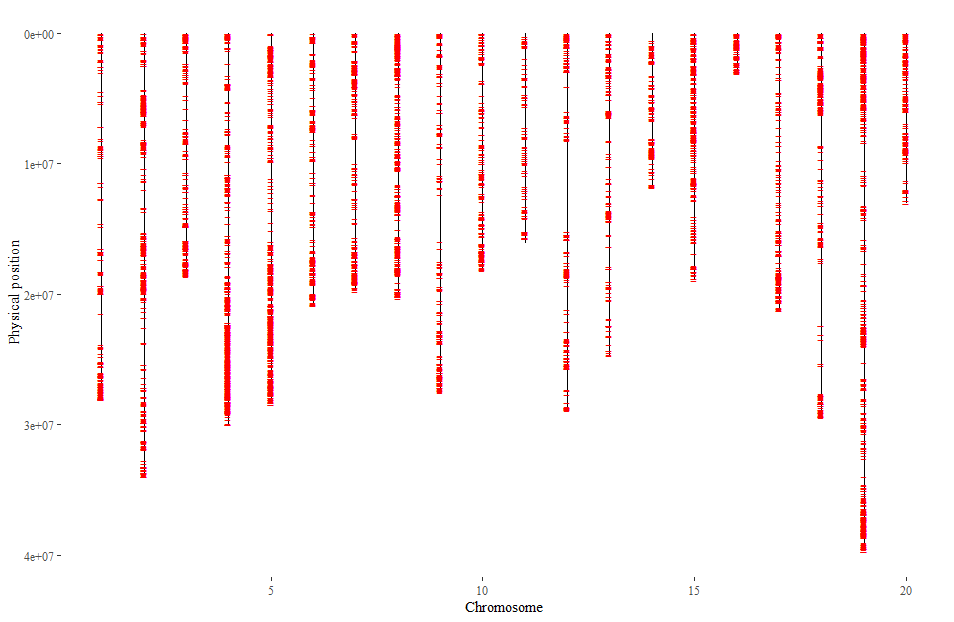


**Fig. S4** Distribution of the 6017 SNPs along the *Dioscorea alata* genetic map.


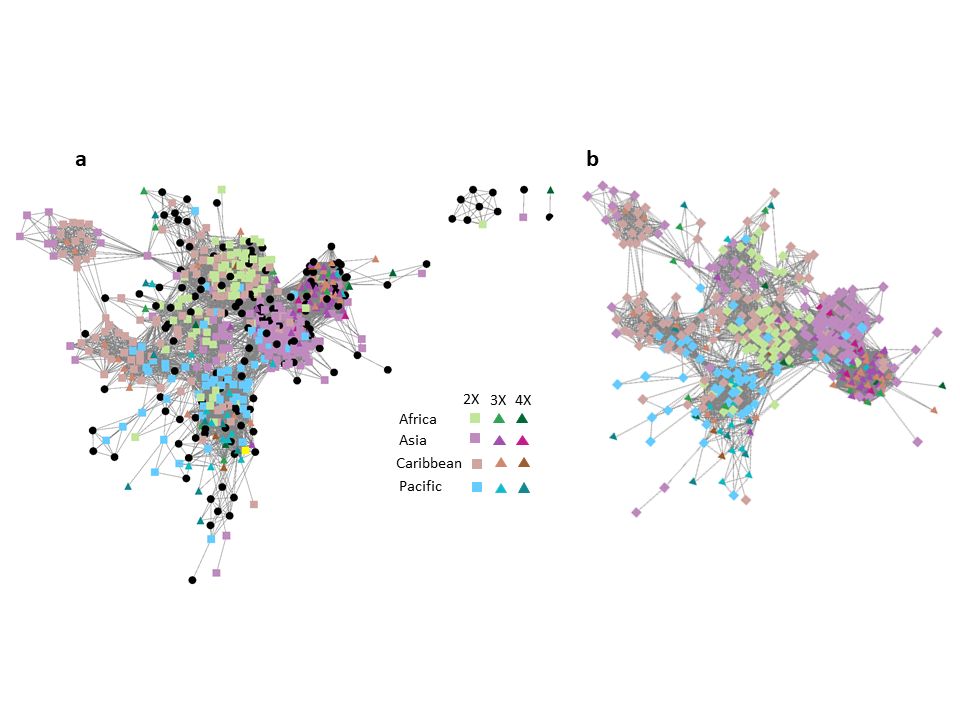


**Fig. S5.** Network visualisation of the genetic relationships between the 643 accessions according to their ploidy level and their geographical origin.

(**a**) The network is based on shared alleles depicting the relationship between accessions with unknown ploidy levels (black nodes) and the others, according to their geographical origins. This figure shows an even distribution of accessions with unknown ploidy. They did not gather according to the ploidy level or genetic gene pool. **(b**) The network shows relationships between diploid, triploid and tetraploid accessions, according to their geographical origin.

**
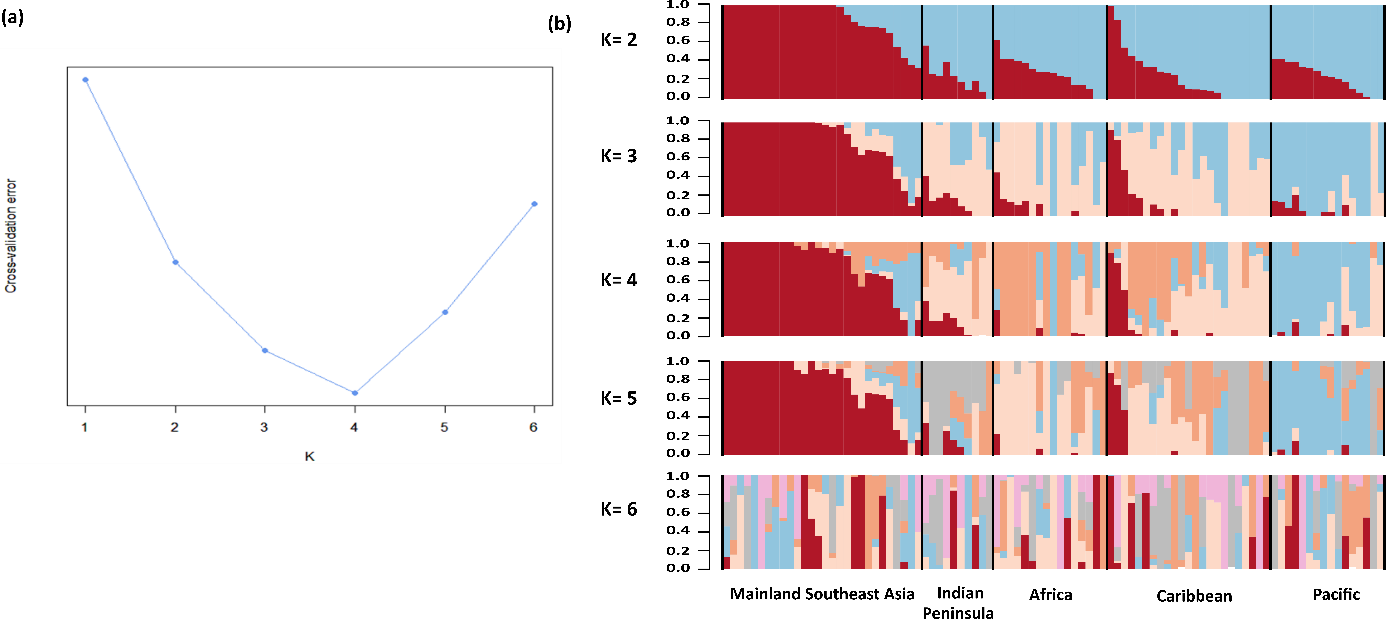
**

**Fig. S6**: Cluster assignment of 93 diploid greater yam genotypes estimated using ADMIXTURE for *K* = 2 to *K* = 6. **a-** Cross validation plot. **b-** Admixture barplot showing the distribution of the *K=2 to 6* genetic clusters

**Table S2** : *Dioscorea alata* accessions used in this study with their geographical origin, name of the provider, measured ploidy, inferred ploidy, MLLs and UGs defined after relatedness analysis, and the membership within each cluster at *K*=4 after ADMIXTURE analysis.

CIRAD: *Centre de Coopération Internationale pour la Recherche Agronomique pour le Développement*; CRB-PT: *Centre de Ressources Biologiques- Plantes Tropicales*; FCRDI: Field Crops Research and Development Institute; NARI: National Agricultural Research Institute; IITA: International Institute of Tropical Agriculture; PRC: Plant Ressources Center; TUA: Tokyo University of Agriculture; Un. Antananarivo: University of Antananarivo ; and VARTC: Vanuatu Agricultural Research and Technical Centre.

| **Accession** | **Country** | **Continent** | **Type** | **Provider** | **Measured Ploidy** | **Inferred Ploidy** | **302 diploids** | **93 diploids** | **Cluster_1** | **Cluster_2** | **Cluster_3** | **Cluster_4** |
| --- | --- | --- | --- | --- | --- | --- | --- | --- | --- | --- | --- | --- |
| Tda4049 | Benin | Africa | Landrace | IITA |  | 2 | MLL12 |  |  |  |  |  |
| Tda4045 | Benin | Africa | Landrace | IITA |  | 2 | UG | UG | 0.00001 | 0.000014 | 0.00001 | 0.999966 |
| Tda1255 | Benin | Africa | Landrace | IITA |  | 3 |  |  |  |  |  |  |
| BGPI-15 | Benin | Africa | Landrace | Cirad |  | 2 | MLL4 |  |  |  |  |  |
| Roujol35 | Benin | Africa | Landrace | Cirad |  | 3 |  |  |  |  |  |  |
| Tda1277 | Benin | Africa | Landrace | IITA |  | 2 | MLL38 | MLL38 | 0.00001 | 0.00001 | 0.00001 | 0.99997 |
| Tda4055 | Benin | Africa | Landrace | IITA |  | NA |  |  |  |  |  |  |
| Tda1188 | Benin | Africa | Landrace | IITA |  | NA |  |  |  |  |  |  |
| Tda3441 | Benin | Africa | Landrace | IITA |  | NA |  |  |  |  |  |  |
| Tda4112 | Burkina faso | Africa | Landrace | IITA |  | NA |  |  |  |  |  |  |
| Tda1366 | Congo | Africa | Landrace | IITA |  | 3 |  |  |  |  |  |  |
| Tda1468 | Cote d'Ivoire | Africa | Landrace | IITA |  | NA |  |  |  |  |  |  |
| Tda3802 | Cote d'Ivoire | Africa | Landrace | IITA |  | 2 | MLL9 |  |  |  |  |  |
| Tda1183 | Cote d'Ivoire | Africa | Landrace | IITA |  | NA |  |  |  |  |  |  |
| Tda1241 | Cote d'Ivoire | Africa | Landrace | IITA |  | 2 | MLL37 |  |  |  |  |  |
| Tda3128 | Equatorial Guinea | Africa | Landrace | IITA |  | NA |  |  |  |  |  |  |
| Tda1367 | Ghana | Africa | Landrace | IITA |  | 3 |  |  |  |  |  |  |
| Tda3278 | Ghana | Africa | Landrace | IITA |  | NA |  |  |  |  |  |  |
| Tda3275 | Ghana | Africa | Landrace | IITA |  | NA |  |  |  |  |  |  |
| Tda1328 | Ghana | Africa | Landrace | IITA |  | 2 | MLL10 |  |  |  |  |  |
| Tda4050 | Ghana | Africa | Landrace | IITA |  | NA |  |  |  |  |  |  |
| BGPI-17 | Ghana | Africa | Landrace | Cirad |  | 2 | UG | UG | 0.00001 | 0.509832 | 0.490148 | 0.00001 |
| Tda3271 | Ghana | Africa | Landrace | IITA |  | 4 |  |  |  |  |  |  |
| Tda2832 | Ghana | Africa | Landrace | IITA |  | 2 | MLL39 |  |  |  |  |  |
| Tda2835 | Ghana | Africa | Landrace | IITA |  | 2 | MLL39 |  |  |  |  |  |
| Tda1224 | Ghana | Africa | Landrace | IITA |  | NA |  |  |  |  |  |  |
| Tda4053 | Ghana | Africa | Landrace | IITA |  | NA |  |  |  |  |  |  |
| Tda4052 | Ghana | Africa | Landrace | IITA |  | 2 | MLL12 |  |  |  |  |  |
| Tda4054 | Ghana | Africa | Landrace | IITA |  | 2 | MLL38 |  |  |  |  |  |
| Tda1247 | Ghana | Africa | Landrace | IITA |  | NA |  |  |  |  |  |  |
| Tda1307 | Ghana | Africa | Landrace | IITA |  | 4 |  |  |  |  |  |  |
| RMM1 | Madagascar | Africa | Landrace | Un. Antananarivo | | 3 |  |  |  |  |  |  |
| RMM2 | Madagascar | Africa | Landrace | Un. Antananarivo | | NA |  |  |  |  |  |  |
| RMM3 | Madagascar | Africa | Landrace | Un. Antananarivo | | 4 |  |  |  |  |  |  |
| RMM4 | Madagascar | Africa | Landrace | Un. Antananarivo | | 2 | UG | UG | 0.00001 | 0.99997 | 0.00001 | 0.00001 |
| RMM5 | Madagascar | Africa | Landrace | Un. Antananarivo | | NA |  |  |  |  |  |  |
| RMM6 | Madagascar | Africa | Landrace | Un. Antananarivo | | NA |  |  |  |  |  |  |
| RMM7 | Madagascar | Africa | Landrace | Un. Antananarivo | | NA |  |  |  |  |  |  |
| RMM9 | Madagascar | Africa | Landrace | Un. Antananarivo | | NA |  |  |  |  |  |  |
| BGPI-22 | Madagascar | Africa | Landrace | Cirad |  | 4 |  |  |  |  |  |  |
| BGPI-16 | Madagascar | Africa | Landrace | Cirad |  | 3 |  |  |  |  |  |  |
| BGPI-12 | Madagascar | Africa | Landrace | Cirad |  | 3 |  |  |  |  |  |  |
| BGPI-19 | Madagascar | Africa | Landrace | Cirad |  | 3 |  |  |  |  |  |  |
| Roujol32 | Madagascar | Africa | Landrace | Cirad |  | 3 |  |  |  |  |  |  |
| Roujol34 | Madagascar | Africa | Landrace | Cirad |  | NA |  |  |  |  |  |  |
| RVV03 | Madagascar | Africa | Landrace | Un. Antananarivo | | 2 | MLL4 |  |  |  |  |  |
| RVV07 | Madagascar | Africa | Landrace | Un. Antananarivo | | NA |  |  |  |  |  |  |
| RVV06 | Madagascar | Africa | Landrace | Un. Antananarivo | | 2 | UG | UG | 0.033726 | 0.560711 | 0.405553 | 0.00001 |
| RVV05 | Madagascar | Africa | Landrace | Un. Antananarivo | | NA |  |  |  |  |  |  |
| RVV08 | Madagascar | Africa | Landrace | Un. Antananarivo | | NA |  |  |  |  |  |  |
| RMM18 | Madagascar | Africa | Landrace | Un. Antananarivo | | NA |  |  |  |  |  |  |
| RMM19 | Madagascar | Africa | Landrace | Un. Antananarivo | | NA |  |  |  |  |  |  |
| RMM12 | Madagascar | Africa | Landrace | Un. Antananarivo | | NA |  |  |  |  |  |  |
| RMM13 | Madagascar | Africa | Landrace | Un. Antananarivo | | NA |  |  |  |  |  |  |
| RMM11 | Madagascar | Africa | Landrace | Un. Antananarivo | | NA |  |  |  |  |  |  |
| RMM16 | Madagascar | Africa | Landrace | Un. Antananarivo | | NA |  |  |  |  |  |  |
| RMM17 | Madagascar | Africa | Landrace | Un. Antananarivo | | 2 | MLL10 |  |  |  |  |  |
| RMM14 | Madagascar | Africa | Landrace | Un. Antananarivo | | 3 |  |  |  |  |  |  |
| RMM25 | Madagascar | Africa | Landrace | Un. Antananarivo | | NA |  |  |  |  |  |  |
| RMM24 | Madagascar | Africa | Landrace | Un. Antananarivo | | NA |  |  |  |  |  |  |
| RMM22 | Madagascar | Africa | Landrace | Un. Antananarivo | | NA |  |  |  |  |  |  |
| RMM21 | Madagascar | Africa | Landrace | Un. Antananarivo | | 2 | MLL33 |  |  |  |  |  |
| Tda1354 | Nigeria | Africa | Landrace | IITA |  | 3 |  |  |  |  |  |  |
| Tda1369 | Nigeria | Africa | Landrace | IITA |  | 4 |  |  |  |  |  |  |
| Tda4061 | Nigeria | Africa | Landrace | IITA |  | NA |  |  |  |  |  |  |
| Tda1466 | Nigeria | Africa | Landrace | IITA |  | 2 | MLL39 | MLL39 | 0.00001 | 0.00001 | 0.00001 | 0.99997 |
| Tda1464 | Nigeria | Africa | Landrace | IITA |  | 2 | MLL36 |  |  |  |  |  |
| CRB25 | Nigeria | Africa | Landrace | CRB-PT | 2 | 2 | MLL7 | MLL7 | 0.00001 | 0.00001 | 0.00001 | 0.99997 |
| Tda1323 | Nigeria | Africa | Landrace | IITA |  | 2 | MLL10 |  |  |  |  |  |
| Tda1273 | Nigeria | Africa | Landrace | IITA |  | NA |  |  |  |  |  |  |
| Tda4059 | Nigeria | Africa | Landrace | IITA |  | 2 | MLL10 |  |  |  |  |  |
| Tda1319 | Nigeria | Africa | Landrace | IITA |  | 4 |  |  |  |  |  |  |
| Tda1318 | Nigeria | Africa | Landrace | IITA |  | NA |  |  |  |  |  |  |
| Tda4063 | Nigeria | Africa | Landrace | IITA |  | NA |  |  |  |  |  |  |
| Tda1348 | Nigeria | Africa | Landrace | IITA |  | NA |  |  |  |  |  |  |
| Tda1342 | Nigeria | Africa | Landrace | IITA |  | NA |  |  |  |  |  |  |
| Tda1351 | Nigeria | Africa | Landrace | IITA |  | 3 |  |  |  |  |  |  |
| Tda1399 | Nigeria | Africa | Landrace | IITA |  | 2 | MLL10 |  |  |  |  |  |
| Tda1394 | Nigeria | Africa | Landrace | IITA |  | NA |  |  |  |  |  |  |
| Tda1286 | Nigeria | Africa | Landrace | IITA |  | NA |  |  |  |  |  |  |
| Tda1276 | Nigeria | Africa | Landrace | IITA |  | 2 | MLL36 |  |  |  |  |  |
| Tda1411 | Nigeria | Africa | Landrace | IITA |  | NA |  |  |  |  |  |  |
| Tda3249 | Nigeria | Africa | Landrace | IITA |  | 2 | MLL4 | MLL4 | 0.00001 | 0.00001 | 0.99997 | 0.00001 |
| Tda1317 | Nigeria | Africa | Landrace | IITA |  | 2 | MLL35 |  |  |  |  |  |
| Tda4126 | Sierra Leone | Africa | Landrace | IITA |  | NA |  |  |  |  |  |  |
| Tda4128 | Sierra Leone | Africa | Landrace | IITA |  | 2 | MLL5 |  |  |  |  |  |
| Tda4125 | Sierra Leone | Africa | Landrace | IITA |  | 4 |  |  |  |  |  |  |
| Tda4130 | Sierra Leone | Africa | Landrace | IITA |  | 2 | MLL37 |  |  |  |  |  |
| Tda4137 | Sierra Leone | Africa | Landrace | IITA |  | 2 | MLL9 | MLL9 | 0.00001 | 0.00001 | 0.00001 | 0.99997 |
| Tda4134 | Sierra Leone | Africa | Landrace | IITA |  | 2 | MLL9 |  |  |  |  |  |
| Tda4138 | Sierra Leone | Africa | Landrace | IITA |  | 2 | MLL10 | MLL10 | 0.078776 | 0.299688 | 0.519663 | 0.101874 |
| Tda4142 | Sierra Leone | Africa | Landrace | IITA |  | NA |  |  |  |  |  |  |
| Tda1061 | Togo | Africa | Landrace | IITA |  | 2 | MLL33 |  |  |  |  |  |
| Tda1145 | Togo | Africa | Landrace | IITA |  | NA |  |  |  |  |  |  |
| Tda1101 | Togo | Africa | Landrace | IITA |  | NA |  |  |  |  |  |  |
| Tda1039 | Togo | Africa | Landrace | IITA |  | 2 | MLL4 |  |  |  |  |  |
| Tda1083 | Togo | Africa | Landrace | IITA |  | NA |  |  |  |  |  |  |
| Tda1082 | Togo | Africa | Landrace | IITA |  | NA |  |  |  |  |  |  |
| Tda1085 | Togo | Africa | Landrace | IITA |  | 2 | MLL10 |  |  |  |  |  |
| Tda1026 | Togo | Africa | Landrace | IITA |  | NA |  |  |  |  |  |  |
| Tda3162 | Togo | Africa | Landrace | IITA |  | 2 | MLL4 |  |  |  |  |  |
| Tda3168 | Togo | Africa | Landrace | IITA |  | NA |  |  |  |  |  |  |
| Tda1134 | Togo | Africa | Landrace | IITA |  | 2 | UG | UG | 0.020338 | 0.615512 | 0.233786 | 0.130364 |
| Tda1005 | Togo | Africa | Landrace | IITA |  | 2 | MLL33 |  |  |  |  |  |
| Tda1004 | Togo | Africa | Landrace | IITA |  | 2 | UG | UG | 0.274251 | 0.00001 | 0.224548 | 0.501191 |
| Tda1006 | Togo | Africa | Landrace | IITA |  | 2 | MLL33 |  |  |  |  |  |
| Tda1049 | Togo | Africa | Landrace | IITA |  | 2 | MLL35 |  |  |  |  |  |
| Tda1311 | Togo | Africa | Landrace | IITA |  | NA |  |  |  |  |  |  |
| Tda1096 | Togo | Africa | Landrace | IITA |  | 2 | MLL36 | MLL36 | 0.00001 | 0.00001 | 0.00001 | 0.99997 |
| Tda1094 | Togo | Africa | Landrace | IITA |  | NA |  |  |  |  |  |  |
| Tda1095 | Togo | Africa | Landrace | IITA |  | 2 | MLL36 |  |  |  |  |  |
| Tda1090 | Togo | Africa | Landrace | IITA |  | NA |  |  |  |  |  |  |
| Tda2868 | Togo | Africa | Landrace | IITA |  | 2 | MLL34 | MLL34 | 0.00001 | 0.00001 | 0.00001 | 0.99997 |
| Tda2869 | Togo | Africa | Landrace | IITA |  | 3 |  |  |  |  |  |  |
| Tda1407 | Togo | Africa | Landrace | IITA |  | 2 | MLL34 |  |  |  |  |  |
| Tda1045 | Togo | Africa | Landrace | IITA |  | 2 | MLL35 | MLL35 | 0.00001 | 0.00001 | 0.00001 | 0.99997 |
| Tda1044 | Togo | Africa | Landrace | IITA |  | 2 | MLL35 |  |  |  |  |  |
| Tda1092b | Togo | Africa | Landrace | IITA |  | 2 | MLL33 |  |  |  |  |  |
| Tda2884 | Togo | Africa | Landrace | IITA |  | 2 | MLL5 |  |  |  |  |  |
| Tda1220 | Togo | Africa | Landrace | IITA |  | 4 |  |  |  |  |  |  |
| Tda2873 | Togo | Africa | Landrace | IITA |  | 2 | MLL4 |  |  |  |  |  |
| Tda1124 | Togo | Africa | Landrace | IITA |  | 3 |  |  |  |  |  |  |
| Tda1010 | Togo | Africa | Landrace | IITA |  | NA |  |  |  |  |  |  |
| Tda1096b | Togo | Africa | Landrace | IITA |  | 2 | MLL36 |  |  |  |  |  |
| Tda4195 | Togo | Africa | Landrace | IITA |  | 2 | MLL12 |  |  |  |  |  |
| Tda3214 | Togo | Africa | Landrace | IITA |  | 2 | MLL9 |  |  |  |  |  |
| Tda1048 | Togo | Africa | Landrace | IITA |  | 2 | MLL35 |  |  |  |  |  |
| Tda1095b | Togo | Africa | Landrace | IITA |  | 2 | MLL36 |  |  |  |  |  |
| Tda1099b | Togo | Africa | Landrace | IITA |  | 2 | MLL33 |  |  |  |  |  |
| Tda1111 | Togo | Africa | Landrace | IITA |  | 4 |  |  |  |  |  |  |
| Tda1118 | Togo | Africa | Landrace | IITA |  | 2 | MLL33 | MLL33 | 0.00001 | 0.223673 | 0.00001 | 0.776307 |
| Tda1027 | Togo | Africa | Landrace | IITA |  | 2 | MLL34 |  |  |  |  |  |
| Tda3205 | Togo | Africa | Landrace | IITA |  | 2 | MLL12 |  |  |  |  |  |
| Tda1028 | Togo | Africa | Landrace | IITA |  | 2 | MLL34 |  |  |  |  |  |
| Tda1022 | Togo | Africa | Landrace | IITA |  | NA |  |  |  |  |  |  |
| Tda1085A | Togo | Africa | Landrace | IITA |  | NA |  |  |  |  |  |  |
| Tda1162 | Togo | Africa | Landrace | IITA |  | 2 | MLL36 |  |  |  |  |  |
| Tda1097 | Togo | Africa | Landrace | IITA |  | 4 |  |  |  |  |  |  |
| Tda1245 | Togo | Africa | Landrace | IITA |  | 2 | MLL37 |  |  |  |  |  |
| Tda3136 | Togo | Africa | Landrace | IITA |  | 2 | MLL5 |  |  |  |  |  |
| Tda1005b | Togo | Africa | Landrace | IITA |  | 2 | MLL33 |  |  |  |  |  |
| 74F-1 | India | Asia | Replicate | Cirad |  | 2 |  |  |  |  |  |  |
| 74F-M | India | Asia | Replicate | Cirad |  | 2 |  |  |  |  |  |  |
| Roujol52 | India | Asia | Landrace | Cirad |  | 2 | UG | UG | 0.093999 | 0.554816 | 0.274513 | 0.076672 |
| Roujol51 | India | Asia | Landrace | Cirad |  | 2 | UG | UG | 0.243628 | 0.45795 | 0.00001 | 0.298412 |
| Roujol56 | India | Asia | Hybrid | Cirad |  | 2 |  |  |  |  |  |  |
| BGPI-21 | India | Asia | Landrace | Cirad |  | 2 | UG | UG | 0.005074 | 0.823461 | 0.00001 | 0.171455 |
| BGPI-25 | India | Asia | Landrace | Cirad |  | 2 | UG | UG | 0.15931 | 0.628153 | 0.00001 | 0.212527 |
| 74F-1bis | India | Asia | Replicate | Cirad |  | 2 |  |  |  |  |  |  |
| 74F-1_4 | India | Asia | Replicate | Cirad |  | 2 |  |  |  |  |  |  |
| Roujol49 | India | Asia | Landrace | Cirad |  | 2 | UG | UG | 0.187721 | 0.548508 | 0.245714 | 0.018058 |
| BGPI-13 | India | Asia | Landrace | Cirad |  | 3 |  |  |  |  |  |  |
| Vua27 | India | Asia | Landrace | VARTC |  | NA |  |  |  |  |  |  |
| Roujol58 | India | Asia | Hybrid | Cirad |  | 2 |  |  |  |  |  |  |
| Roujol53 | India | Asia | Landrace | Cirad | 2 | 2 | UG | UG | 0.006794 | 0.797291 | 0.195905 | 0.00001 |
| 14M-M | India | Asia | Landrace | Cirad | 2 | 2 | MLL1 |  |  |  |  |  |
| Roujol74 | India | Asia | Landrace | Cirad |  | 2 | UG | UG | 0.00001 | 0.99997 | 0.00001 | 0.00001 |
| Vua33 | India | Asia | Landrace | VARTC |  | 3 |  |  |  |  |  |  |
| 74F-1_3 | India | Asia | Landrace | Cirad |  | NA |  |  |  |  |  |  |
| Vua58 | India | Asia | Landrace | VARTC |  | 2 | UG | UG | 0.369889 | 0.150595 | 0.005246 | 0.474271 |
| Vua51 | India | Asia | Landrace | VARTC |  | NA |  |  |  |  |  |  |
| Vua23a | India | Asia | Landrace | VARTC |  | 2 | MLL19 |  |  |  |  |  |
| Roujol60 | India | Asia | Hybrid | Cirad |  | 2 |  |  |  |  |  |  |
| 74F-2_3 | India | Asia | Replicate | Cirad |  | 2 |  |  |  |  |  |  |
| 14M-Mbis | India | Asia | Landrace | Cirad | 2 | 2 | MLL1 | MLL1 | 0.157212 | 0.69489 | 0.00001 | 0.147888 |
| 74F-2bis | India | Asia | Replicate | Cirad |  | 2 |  |  |  |  |  |  |
| A-98 | Japan | Asia | Landrace | TUA |  | 2 | UG | UG | 0.00001 | 0.00001 | 0.883969 | 0.116011 |
| A-50 | Japan | Asia | Landrace | TUA |  | 2 | MLL2 |  |  |  |  |  |
| A-133 | Japan | Asia | Landrace | TUA |  | 2 | UG | UG | 0.169939 | 0.201291 | 0.515171 | 0.1136 |
| A-132 | Japan | Asia | Landrace | TUA |  | NA |  |  |  |  |  |  |
| A-45 | Japan | Asia | Landrace | TUA |  | 3 |  |  |  |  |  |  |
| ETH13 | Sri Lanka | Asia | Landrace | FCRDI |  | 2 | MLL14 |  |  |  |  |  |
| ETH12 | Sri Lanka | Asia | Landrace | FCRDI |  | NA |  |  |  |  |  |  |
| ETH10 | Sri Lanka | Asia | Landrace | FCRDI |  | NA |  |  |  |  |  |  |
| ETH17 | Sri Lanka | Asia | Landrace | FCRDI |  | 2 | MLL9 |  |  |  |  |  |
| ETH11 | Sri Lanka | Asia | Landrace | FCRDI |  | NA |  |  |  |  |  |  |
| ETH14 | Sri Lanka | Asia | Landrace | FCRDI |  | NA |  |  |  |  |  |  |
| ETH16 | Sri Lanka | Asia | Landrace | FCRDI |  | NA |  |  |  |  |  |  |
| ETH15 | Sri Lanka | Asia | Landrace | FCRDI |  | 2 | MLL9 |  |  |  |  |  |
| ETH01 | Sri Lanka | Asia | Landrace | FCRDI |  | NA |  |  |  |  |  |  |
| ETH41 | Sri Lanka | Asia | Landrace | FCRDI |  | NA |  |  |  |  |  |  |
| ETH42 | Sri Lanka | Asia | Landrace | FCRDI |  | 2 | MLL14 |  |  |  |  |  |
| ETH39 | Sri Lanka | Asia | Landrace | FCRDI |  | 3 |  |  |  |  |  |  |
| ETH35 | Sri Lanka | Asia | Landrace | FCRDI |  | NA |  |  |  |  |  |  |
| ETH34 | Sri Lanka | Asia | Landrace | FCRDI |  | 3 |  |  |  |  |  |  |
| ETH37 | Sri Lanka | Asia | Landrace | FCRDI |  | 3 |  |  |  |  |  |  |
| ETH31 | Sri Lanka | Asia | Landrace | FCRDI |  | NA |  |  |  |  |  |  |
| ETH33 | Sri Lanka | Asia | Landrace | FCRDI |  | 3 |  |  |  |  |  |  |
| ETH29 | Sri Lanka | Asia | Landrace | FCRDI |  | 3 |  |  |  |  |  |  |
| ETH25 | Sri Lanka | Asia | Landrace | FCRDI |  | NA |  |  |  |  |  |  |
| ETH20 | Sri Lanka | Asia | Landrace | FCRDI |  | 2 | UG | UG | 0.00001 | 0.182546 | 0.00001 | 0.817434 |
| ETH21 | Sri Lanka | Asia | Landrace | FCRDI |  | NA |  |  |  |  |  |  |
| PRC176 | Vietnam | Asia | Landrace | PRC |  | 2 | MLL21 |  |  |  |  |  |
| PRC16 | Vietnam | Asia | Landrace | PRC |  | 3 |  |  |  |  |  |  |
| PRC14 | Vietnam | Asia | Landrace | PRC |  | 3 |  |  |  |  |  |  |
| PRC13 | Vietnam | Asia | Landrace | PRC |  | 3 |  |  |  |  |  |  |
| PRC82 | Vietnam | Asia | Landrace | PRC |  | 3 |  |  |  |  |  |  |
| PRC86 | Vietnam | Asia | Landrace | PRC |  | 2 | MLL32 |  |  |  |  |  |
| PRC5 | Vietnam | Asia | Landrace | PRC |  | 3 |  |  |  |  |  |  |
| PRC148 | Vietnam | Asia | Landrace | PRC |  | 2 | MLL20 | MLL20 | 0.93774 | 0.00001 | 0.00001 | 0.06224 |
| PRC145 | Vietnam | Asia | Landrace | PRC |  | NA |  |  |  |  |  |  |
| PRC146 | Vietnam | Asia | Landrace | PRC |  | 2 | MLL32 | MLL32 | 0.661016 | 0.00001 | 0.00001 | 0.338964 |
| PRC147 | Vietnam | Asia | Landrace | PRC |  | 3 |  |  |  |  |  |  |
| PRC141 | Vietnam | Asia | Landrace | PRC |  | 3 |  |  |  |  |  |  |
| PRC248 | Vietnam | Asia | Landrace | PRC |  | NA |  |  |  |  |  |  |
| PRC247 | Vietnam | Asia | Landrace | PRC |  | 2 | MLL29 | MLL29 | 0.526831 | 0.00001 | 0.00001 | 0.473149 |
| PRC246 | Vietnam | Asia | Landrace | PRC |  | 2 | MLL29 |  |  |  |  |  |
| PRC245 | Vietnam | Asia | Landrace | PRC |  | 2 | MLL28 |  |  |  |  |  |
| PRC244 | Vietnam | Asia | Landrace | PRC |  | 2 | MLL24 |  |  |  |  |  |
| PRC243 | Vietnam | Asia | Landrace | PRC |  | NA |  |  |  |  |  |  |
| PRC242 | Vietnam | Asia | Landrace | PRC |  | 2 | MLL18 |  |  |  |  |  |
| PRC241 | Vietnam | Asia | Landrace | PRC |  | 2 | MLL18 |  |  |  |  |  |
| PRC228 | Vietnam | Asia | Landrace | PRC |  | 2 | MLL27 |  |  |  |  |  |
| PRC2 | Vietnam | Asia | Landrace | PRC |  | NA |  |  |  |  |  |  |
| PRC1 | Vietnam | Asia | Landrace | PRC |  | 2 | MLL17 |  |  |  |  |  |
| PRC7 | Vietnam | Asia | Landrace | PRC |  | NA |  |  |  |  |  |  |
| PRC202 | Vietnam | Asia | Landrace | PRC |  | 3 |  |  |  |  |  |  |
| PRC201 | Vietnam | Asia | Landrace | PRC |  | 2 | MLL25 |  |  |  |  |  |
| PRC200 | Vietnam | Asia | Landrace | PRC |  | 2 | MLL23 | MLL23 | 0.932955 | 0.067025 | 0.00001 | 0.00001 |
| PRC207 | Vietnam | Asia | Landrace | PRC |  | 2 | UG | UG | 0.99997 | 0.00001 | 0.00001 | 0.00001 |
| PRC206 | Vietnam | Asia | Landrace | PRC |  | 2 | MLL25 |  |  |  |  |  |
| PRC205 | Vietnam | Asia | Landrace | PRC |  | 2 | UG | UG | 0.99997 | 0.00001 | 0.00001 | 0.00001 |
| PRC204 | Vietnam | Asia | Landrace | PRC |  | 3 |  |  |  |  |  |  |
| PRC209 | Vietnam | Asia | Landrace | PRC |  | 2 | MLL8 |  |  |  |  |  |
| PRC112 | Vietnam | Asia | Landrace | PRC |  | NA |  |  |  |  |  |  |
| PRC111 | Vietnam | Asia | Landrace | PRC |  | 3 |  |  |  |  |  |  |
| PRC110 | Vietnam | Asia | Landrace | PRC |  | 3 |  |  |  |  |  |  |
| PRC290 | Vietnam | Asia | Landrace | PRC |  | 2 | MLL14 |  |  |  |  |  |
| PRC291 | Vietnam | Asia | Landrace | PRC |  | NA |  |  |  |  |  |  |
| PRC119 | Vietnam | Asia | Landrace | PRC |  | 3 |  |  |  |  |  |  |
| PRC276 | Vietnam | Asia | Landrace | PRC |  | NA |  |  |  |  |  |  |
| PRC277 | Vietnam | Asia | Landrace | PRC |  | 2 | MLL19 |  |  |  |  |  |
| PRC274 | Vietnam | Asia | Landrace | PRC |  | NA |  |  |  |  |  |  |
| PRC275 | Vietnam | Asia | Landrace | PRC |  | 2 | UG | UG | 0.658061 | 0.007501 | 0.078709 | 0.255729 |
| PRC272 | Vietnam | Asia | Landrace | PRC |  | NA |  |  |  |  |  |  |
| PRC270 | Vietnam | Asia | Landrace | PRC |  | 2 | MLL31 | MLL31 | 0.99997 | 0.00001 | 0.00001 | 0.00001 |
| PRC271 | Vietnam | Asia | Landrace | PRC |  | 2 | MLL21 |  |  |  |  |  |
| PRC15 | Vietnam | Asia | Landrace | PRC |  | NA |  |  |  |  |  |  |
| PRC221 | Vietnam | Asia | Landrace | PRC |  | 3 |  |  |  |  |  |  |
| PRC283 | Vietnam | Asia | Landrace | PRC |  | 2 | MLL31 |  |  |  |  |  |
| PRC74 | Vietnam | Asia | Landrace | PRC |  | 2 | MLL27 |  |  |  |  |  |
| PRC77 | Vietnam | Asia | Landrace | PRC |  | 2 | MLL17 | MLL17 | 0.915066 | 0.00001 | 0.00001 | 0.084914 |
| PRC76 | Vietnam | Asia | Landrace | PRC |  | 2 | MLL30 |  |  |  |  |  |
| PRC70 | Vietnam | Asia | Landrace | PRC |  | 3 |  |  |  |  |  |  |
| PRC72 | Vietnam | Asia | Landrace | PRC |  | NA |  |  |  |  |  |  |
| PRC128 | Vietnam | Asia | Landrace | PRC |  | 2 | MLL19 |  |  |  |  |  |
| PRC126 | Vietnam | Asia | Landrace | PRC |  | 2 | MLL19 |  |  |  |  |  |
| PRC121 | Vietnam | Asia | Landrace | PRC |  | 2 | MLL18 |  |  |  |  |  |
| PRC153 | Vietnam | Asia | Landrace | PRC |  | 3 |  |  |  |  |  |  |
| PRC151 | Vietnam | Asia | Landrace | PRC |  | 2 | MLL20 |  |  |  |  |  |
| PRC150 | Vietnam | Asia | Landrace | PRC |  | 2 | MLL20 |  |  |  |  |  |
| PRC155 | Vietnam | Asia | Landrace | PRC |  | 2 | MLL21 |  |  |  |  |  |
| PRC239 | Vietnam | Asia | Landrace | PRC |  | 2 | MLL21 |  |  |  |  |  |
| PRC232 | Vietnam | Asia | Landrace | PRC |  | 2 | MLL23 |  |  |  |  |  |
| PRC233 | Vietnam | Asia | Landrace | PRC |  | 2 | MLL24 |  |  |  |  |  |
| PRC230 | Vietnam | Asia | Landrace | PRC |  | 3 |  |  |  |  |  |  |
| PRC231 | Vietnam | Asia | Landrace | PRC |  | 2 | MLL14 |  |  |  |  |  |
| PRC236 | Vietnam | Asia | Landrace | PRC |  | 2 | MLL14 |  |  |  |  |  |
| PRC237 | Vietnam | Asia | Landrace | PRC |  | 2 | MLL14 |  |  |  |  |  |
| PRC234 | Vietnam | Asia | Landrace | PRC |  | 2 | UG | UG | 0.642798 | 0.04479 | 0.134278 | 0.178135 |
| PRC235 | Vietnam | Asia | Landrace | PRC |  | NA |  |  |  |  |  |  |
| PRC31 | Vietnam | Asia | Landrace | PRC |  | NA |  |  |  |  |  |  |
| PRC280 | Vietnam | Asia | Landrace | PRC |  | 2 | MLL21 |  |  |  |  |  |
| PRC37 | Vietnam | Asia | Landrace | PRC |  | 2 | MLL19 |  |  |  |  |  |
| PRC38 | Vietnam | Asia | Landrace | PRC |  | 3 |  |  |  |  |  |  |
| PRC238 | Vietnam | Asia | Landrace | PRC |  | 2 | MLL20 |  |  |  |  |  |
| PRC81 | Vietnam | Asia | Landrace | PRC |  | 2 | MLL24 |  |  |  |  |  |
| PRC88 | Vietnam | Asia | Landrace | PRC |  | 2 | MLL14 |  |  |  |  |  |
| PRC162 | Vietnam | Asia | Landrace | PRC |  | 2 | MLL22 |  |  |  |  |  |
| PRC163 | Vietnam | Asia | Landrace | PRC |  | 2 | MLL8 |  |  |  |  |  |
| PRC289 | Vietnam | Asia | Landrace | PRC |  | 2 | UG | UG | 0.300352 | 0.00001 | 0.464958 | 0.234681 |
| PRC288 | Vietnam | Asia | Landrace | PRC |  | 2 | MLL14 | MLL14 | 0.17142 | 0.086621 | 0.540502 | 0.201457 |
| PRC166 | Vietnam | Asia | Landrace | PRC |  | 2 | MLL8 | MLL8 | 0.99997 | 0.00001 | 0.00001 | 0.00001 |
| PRC167 | Vietnam | Asia | Landrace | PRC |  | NA |  |  |  |  |  |  |
| PRC165 | Vietnam | Asia | Landrace | PRC |  | 2 | MLL18 |  |  |  |  |  |
| PRC282 | Vietnam | Asia | Landrace | PRC |  | 2 | MLL23 |  |  |  |  |  |
| PRC281 | Vietnam | Asia | Landrace | PRC |  | 2 | MLL2 | MLL2 | 0.99997 | 0.00001 | 0.00001 | 0.00001 |
| PRC287 | Vietnam | Asia | Landrace | PRC |  | 2 | MLL27 | MLL27 | 0.851609 | 0.015277 | 0.040022 | 0.093091 |
| PRC286 | Vietnam | Asia | Landrace | PRC |  | 3 |  |  |  |  |  |  |
| PRC285 | Vietnam | Asia | Landrace | PRC |  | 2 | MLL14 |  |  |  |  |  |
| PRC284 | Vietnam | Asia | Landrace | PRC |  | 2 | MLL8 |  |  |  |  |  |
| PRC265 | Vietnam | Asia | Landrace | PRC |  | 4 |  |  |  |  |  |  |
| PRC264 | Vietnam | Asia | Landrace | PRC |  | NA |  |  |  |  |  |  |
| PRC267 | Vietnam | Asia | Landrace | PRC |  | 2 | MLL29 |  |  |  |  |  |
| PRC266 | Vietnam | Asia | Landrace | PRC |  | 2 | MLL23 |  |  |  |  |  |
| PRC261 | Vietnam | Asia | Landrace | PRC |  | 2 | MLL30 |  |  |  |  |  |
| PRC260 | Vietnam | Asia | Landrace | PRC |  | 2 | MLL23 |  |  |  |  |  |
| PRC263 | Vietnam | Asia | Landrace | PRC |  | 4 |  |  |  |  |  |  |
| PRC262 | Vietnam | Asia | Landrace | PRC |  | 2 | MLL29 |  |  |  |  |  |
| PRC269 | Vietnam | Asia | Landrace | PRC |  | 2 | MLL21 |  |  |  |  |  |
| PRC268 | Vietnam | Asia | Landrace | PRC |  | 2 | MLL21 | MLL21 | 0.885786 | 0.00001 | 0.00001 | 0.114194 |
| PRC59 | Vietnam | Asia | Landrace | PRC |  | 2 | MLL14 |  |  |  |  |  |
| PRC63 | Vietnam | Asia | Landrace | PRC |  | NA |  |  |  |  |  |  |
| PRC69 | Vietnam | Asia | Landrace | PRC |  | 2 | UG | UG | 0.99997 | 0.00001 | 0.00001 | 0.00001 |
| PRC67 | Vietnam | Asia | Landrace | PRC |  | 3 |  |  |  |  |  |  |
| PRC65 | Vietnam | Asia | Landrace | PRC |  | 3 |  |  |  |  |  |  |
| PRC135 | Vietnam | Asia | Landrace | PRC |  | 2 | MLL27 |  |  |  |  |  |
| PRC134 | Vietnam | Asia | Landrace | PRC |  | NA |  |  |  |  |  |  |
| PRC130 | Vietnam | Asia | Landrace | PRC |  | 2 | MLL14 |  |  |  |  |  |
| PRC132 | Vietnam | Asia | Landrace | PRC |  | 2 | MLL8 |  |  |  |  |  |
| PRC292 | Vietnam | Asia | Landrace | PRC |  | 2 | MLL21 |  |  |  |  |  |
| PRC229 | Vietnam | Asia | Landrace | PRC |  | 2 | MLL19 |  |  |  |  |  |
| PRC181 | Vietnam | Asia | Landrace | PRC |  | 2 | MLL24 |  |  |  |  |  |
| PRC182 | Vietnam | Asia | Landrace | PRC |  | 3 |  |  |  |  |  |  |
| PRC183 | Vietnam | Asia | Landrace | PRC |  | 3 |  |  |  |  |  |  |
| PRC184 | Vietnam | Asia | Landrace | PRC |  | NA |  |  |  |  |  |  |
| PRC186 | Vietnam | Asia | Landrace | PRC |  | NA |  |  |  |  |  |  |
| PRC187 | Vietnam | Asia | Landrace | PRC |  | 2 | UG | UG | 0.99997 | 0.00001 | 0.00001 | 0.00001 |
| PRC188 | Vietnam | Asia | Landrace | PRC |  | NA |  |  |  |  |  |  |
| PRC189 | Vietnam | Asia | Landrace | PRC |  | 3 |  |  |  |  |  |  |
| PRC223 | Vietnam | Asia | Landrace | PRC |  | NA |  |  |  |  |  |  |
| PRC222 | Vietnam | Asia | Landrace | PRC |  | 3 |  |  |  |  |  |  |
| PRC225 | Vietnam | Asia | Landrace | PRC |  | 3 |  |  |  |  |  |  |
| PRC224 | Vietnam | Asia | Landrace | PRC |  | 3 |  |  |  |  |  |  |
| PRC227 | Vietnam | Asia | Landrace | PRC |  | NA |  |  |  |  |  |  |
| PRC226 | Vietnam | Asia | Landrace | PRC |  | NA |  |  |  |  |  |  |
| PRC22 | Vietnam | Asia | Landrace | PRC |  | 2 | MLL26 |  |  |  |  |  |
| PRC21 | Vietnam | Asia | Landrace | PRC |  | 2 | MLL19 |  |  |  |  |  |
| PRC26 | Vietnam | Asia | Landrace | PRC |  | 3 |  |  |  |  |  |  |
| PRC24 | Vietnam | Asia | Landrace | PRC |  | NA |  |  |  |  |  |  |
| PRC25 | Vietnam | Asia | Landrace | PRC |  | 2 | MLL25 |  |  |  |  |  |
| PRC97 | Vietnam | Asia | Landrace | PRC |  | 2 | MLL22 | MLL22 | 0.99997 | 0.00001 | 0.00001 | 0.00001 |
| PRC93 | Vietnam | Asia | Landrace | PRC |  | 2 | MLL21 |  |  |  |  |  |
| PRC92 | Vietnam | Asia | Landrace | PRC |  | NA |  |  |  |  |  |  |
| PRC90 | Vietnam | Asia | Landrace | PRC |  | 4 |  |  |  |  |  |  |
| PRC99 | Vietnam | Asia | Landrace | PRC |  | 2 | MLL24 | MLL24 | 0.962426 | 0.00001 | 0.00001 | 0.037554 |
| PRC214 | Vietnam | Asia | Landrace | PRC |  | NA |  |  |  |  |  |  |
| PRC3 | Vietnam | Asia | Landrace | PRC |  | NA |  |  |  |  |  |  |
| PRC170 | Vietnam | Asia | Landrace | PRC |  | 2 | MLL14 |  |  |  |  |  |
| PRC173 | Vietnam | Asia | Landrace | PRC |  | 2 | MLL23 |  |  |  |  |  |
| PRC172 | Vietnam | Asia | Landrace | PRC |  | 2 | MLL21 |  |  |  |  |  |
| PRC174 | Vietnam | Asia | Landrace | PRC |  | 2 | MLL23 |  |  |  |  |  |
| PRC177 | Vietnam | Asia | Landrace | PRC |  | NA |  |  |  |  |  |  |
| PRC218 | Vietnam | Asia | Landrace | PRC |  | 2 | MLL14 |  |  |  |  |  |
| PRC250 | Vietnam | Asia | Landrace | PRC |  | 4 |  |  |  |  |  |  |
| PRC253 | Vietnam | Asia | Landrace | PRC |  | 3 |  |  |  |  |  |  |
| PRC254 | Vietnam | Asia | Landrace | PRC |  | 2 | UG | UG | 0.99997 | 0.00001 | 0.00001 | 0.00001 |
| PRC258 | Vietnam | Asia | Landrace | PRC |  | 2 | MLL24 |  |  |  |  |  |
| PRC259 | Vietnam | Asia | Landrace | PRC |  | 2 | MLL28 | MLL28 | 0.941645 | 0.058335 | 0.00001 | 0.00001 |
| PRC30 | Vietnam | Asia | Landrace | PRC |  | 4 |  |  |  |  |  |  |
| PRC56 | Vietnam | Asia | Landrace | PRC |  | 3 |  |  |  |  |  |  |
| PRC108 | Vietnam | Asia | Landrace | PRC |  | 3 |  |  |  |  |  |  |
| PRC109 | Vietnam | Asia | Landrace | PRC |  | 3 |  |  |  |  |  |  |
| PRC102 | Vietnam | Asia | Landrace | PRC |  | 3 |  |  |  |  |  |  |
| PRC105 | Vietnam | Asia | Landrace | PRC |  | 2 | MLL14 |  |  |  |  |  |
| PRC106 | Vietnam | Asia | Landrace | PRC |  | 2 | MLL19 |  |  |  |  |  |
| PRC216 | Vietnam | Asia | Landrace | PRC |  | 2 | MLL19 |  |  |  |  |  |
| PRC217 | Vietnam | Asia | Landrace | PRC |  | 2 | MLL21 |  |  |  |  |  |
| PRC210 | Vietnam | Asia | Landrace | PRC |  | NA |  |  |  |  |  |  |
| PRC211 | Vietnam | Asia | Landrace | PRC |  | 4 |  |  |  |  |  |  |
| PRC212 | Vietnam | Asia | Landrace | PRC |  | 2 | MLL19 |  |  |  |  |  |
| PRC213 | Vietnam | Asia | Landrace | PRC |  | 2 | MLL26 | MLL26 | 0.99997 | 0.00001 | 0.00001 | 0.00001 |
| PRC219 | Vietnam | Asia | Landrace | PRC |  | 2 | UG | UG | 0.672085 | 0.030155 | 0.14602 | 0.15174 |
| PRC199 | Vietnam | Asia | Landrace | PRC |  | 2 | MLL2 |  |  |  |  |  |
| PRC198 | Vietnam | Asia | Landrace | PRC |  | NA |  |  |  |  |  |  |
| PRC197 | Vietnam | Asia | Landrace | PRC |  | 2 | MLL21 |  |  |  |  |  |
| PRC196 | Vietnam | Asia | Landrace | PRC |  | 2 | MLL2 |  |  |  |  |  |
| PRC195 | Vietnam | Asia | Landrace | PRC |  | 2 | MLL30 | MLL30 | 0.60381 | 0.074136 | 0.125808 | 0.196247 |
| PRC194 | Vietnam | Asia | Landrace | PRC |  | 4 |  |  |  |  |  |  |
| PRC193 | Vietnam | Asia | Landrace | PRC |  | 2 | MLL19 |  |  |  |  |  |
| PRC192 | Vietnam | Asia | Landrace | PRC |  | 3 |  |  |  |  |  |  |
| PRC191 | Vietnam | Asia | Landrace | PRC |  | 2 | MLL19 | MLL19 | 0.99997 | 0.00001 | 0.00001 | 0.00001 |
| PRC190 | Vietnam | Asia | Landrace | PRC |  | NA |  |  |  |  |  |  |
| CRB57 | Brazil | Caribbean | Landrace | CRB-PT |  | 2 | MLL12 | MLL12 | 0.00001 | 0.084893 | 0.316674 | 0.598423 |
| BGPI-18 | Cuba | Caribbean | Landrace | Cirad |  | 2 | MLL5 |  |  |  |  |  |
| Roujol36 | Cuba | Caribbean | Landrace | Cirad |  | 3 |  |  |  |  |  |  |
| BGPI-30 | Cuba | Caribbean | Landrace | Cirad |  | 2 | UG | UG | 0.00001 | 0.854529 | 0.145451 | 0.00001 |
| G58 | Dominican Republic | Caribbean | Landrace | CRB-PT |  | 2 | MLL4 |  |  |  |  |  |
| CRB60 | Dominican Republic | Caribbean | Landrace | CRB-PT |  | 2 | MLL4 |  |  |  |  |  |
| CRB58 | Dominican Republic | Caribbean | Landrace | CRB-PT |  | 2 | MLL4 |  |  |  |  |  |
| CRB56 | French Guyana | Caribbean | Landrace | CRB-PT | 3 | 3 |  |  |  |  |  |  |
| CRB615 | French Guyana | Caribbean | Landrace | CRB-PT |  | 2 | UG | UG | 0.00001 | 0.877812 | 0.061081 | 0.061096 |
| CRB38 | French Guyana | Caribbean | Landrace | CRB-PT |  | NA |  |  |  |  |  |  |
| G27 | Guadeloupe | Caribbean | Landrace | CRB-PT |  | 2 | MLL15 |  |  |  |  |  |
| CRB79 | Guadeloupe | Caribbean | Landrace | CRB-PT |  | 2 | MLL9 |  |  |  |  |  |
| CRB760 | Guadeloupe | Caribbean | Hybrid | CRB-PT |  | NA |  |  |  |  |  |  |
| CRB428 | Guadeloupe | Caribbean | Hybrid | CRB-PT |  | 2 |  |  |  |  |  |  |
| CRB27 | Guadeloupe | Caribbean | Landrace | CRB-PT |  | 2 | MLL8 |  |  |  |  |  |
| G402 | Guadeloupe | Caribbean | Landrace | CRB-PT |  | NA |  |  |  |  |  |  |
| Roujol44 | Guadeloupe | Caribbean | Landrace | Cirad |  | 2 | MLL13 |  |  |  |  |  |
| Roujol43 | Guadeloupe | Caribbean | Landrace | Cirad |  | 2 | UG | UG | 0.00001 | 0.99997 | 0.00001 | 0.00001 |
| Roujol42 | Guadeloupe | Caribbean | Landrace | Cirad |  | 2 | UG | UG | 0.00001 | 0.530255 | 0.025965 | 0.44377 |
| CRB87 | Guadeloupe | Caribbean | Landrace | CRB-PT |  | 3 |  |  |  |  |  |  |
| KABUSA-2_3 | Guadeloupe | Caribbean | Landrace | Cirad |  | 3 |  |  |  |  |  |  |
| Roujol75 | Guadeloupe | Caribbean | Landrace | Cirad |  | 2 | UG | UG | 0.487675 | 0.512305 | 0.00001 | 0.00001 |
| KABUSA-1_4 | Guadeloupe | Caribbean | Landrace | Cirad |  | 2 | UG | UG | 0.00001 | 0.295771 | 0.704209 | 0.00001 |
| KABUSA-2 | Guadeloupe | Caribbean | Landrace | Cirad |  | 2 | MLL13 |  |  |  |  |  |
| KABUSA-M | Guadeloupe | Caribbean | Landrace | Cirad |  | 2 | MLL13 |  |  |  |  |  |
| CRB390 | Guadeloupe | Caribbean | Landrace | CRB-PT |  | 2 | UG | UG | 0.00001 | 0.490847 | 0.509133 | 0.00001 |
| G36 | Guadeloupe | Caribbean | Landrace | CRB-PT |  | 3 |  |  |  |  |  |  |
| G87 | Guadeloupe | Caribbean | Landrace | CRB-PT |  | NA |  |  |  |  |  |  |
| CRB475 | Guadeloupe | Caribbean | Hybrid | CRB-PT |  | 2 |  |  |  |  |  |  |
| CRB403 | Guadeloupe | Caribbean | Landrace | CRB-PT | 2 | 2 | MLL5 | MLL5 | 0.02671 | 0.738381 | 0.234898 | 0.00001 |
| CRB758 | Guadeloupe | Caribbean | Hybrid | CRB-PT |  | NA |  |  |  |  |  |  |
| CRB757 | Guadeloupe | Caribbean | Landrace | CRB-PT |  | 2 | UG | UG | 0.00001 | 0.511615 | 0.00001 | 0.488365 |
| CRB32 | Guadeloupe | Caribbean | Landrace | CRB-PT |  | 2 | MLL11 |  |  |  |  |  |
| CRB36 | Guadeloupe | Caribbean | Landrace | CRB-PT | 3 | 3 |  |  |  |  |  |  |
| CRB97 | Haiti | Caribbean | Landrace | CRB-PT | 3 | 3 |  |  |  |  |  |  |
| CRB429 | Haiti | Caribbean | Landrace | CRB-PT |  | 2 | MLL9 |  |  |  |  |  |
| BGPI-6 | Haiti | Caribbean | Landrace | Cirad |  | 3 |  |  |  |  |  |  |
| CRB62 | Haiti | Caribbean | Landrace | CRB-PT |  | 2 | MLL9 |  |  |  |  |  |
| CRB63 | Haiti | Caribbean | Landrace | CRB-PT | 3 | 3 |  |  |  |  |  |  |
| CRB67 | Haiti | Caribbean | Landrace | CRB-PT |  | 2 | UG | UG | 0.00001 | 0.687706 | 0.167808 | 0.144477 |
| Roujol38 | Haiti | Caribbean | Landrace | Cirad |  | 2 | MLL18 | MLL18 | 0.885247 | 0.00001 | 0.00001 | 0.114733 |
| CRB110 | Haiti | Caribbean | Landrace | CRB-PT | 3 | 3 |  |  |  |  |  |  |
| G110 | Haiti | Caribbean | Landrace | CRB-PT |  | 3 |  |  |  |  |  |  |
| G429 | Haiti | Caribbean | Landrace | CRB-PT |  | 2 | MLL9 |  |  |  |  |  |
| CRB47 | Haiti | Caribbean | Landrace | CRB-PT |  | 2 | UG | UG | 0.064454 | 0.128498 | 0.093472 | 0.713575 |
| G62 | Haiti | Caribbean | Landrace | CRB-PT |  | 2 | MLL9 |  |  |  |  |  |
| G63 | Haiti | Caribbean | Landrace | CRB-PT |  | 2 | MLL12 |  |  |  |  |  |
| G52 | Jamaica | Caribbean | Landrace | CRB-PT |  | 3 |  |  |  |  |  |  |
| CRB94 | Jamaica | Caribbean | Landrace | CRB-PT |  | 3 |  |  |  |  |  |  |
| CRB52 | Jamaica | Caribbean | Landrace | CRB-PT |  | 3 |  |  |  |  |  |  |
| CRB107 | Martinique | Caribbean | Landrace | CRB-PT |  | 2 | MLL6 | MLL6 | 0.00001 | 0.00001 | 0.172965 | 0.827015 |
| CRB64 | Martinique | Caribbean | Landrace | CRB-PT |  | 2 | MLL9 |  |  |  |  |  |
| CRB82 | Martinique | Caribbean | Landrace | CRB-PT | 3 | 3 |  |  |  |  |  |  |
| CRB80 | Martinique | Caribbean | Landrace | CRB-PT | 3 | 3 |  |  |  |  |  |  |
| CRB89 | Martinique | Caribbean | Landrace | CRB-PT |  | 3 |  |  |  |  |  |  |
| CRB01 | Martinique | Caribbean | Landrace | CRB-PT |  | 2 | MLL8 |  |  |  |  |  |
| CRB91 | Martinique | Caribbean | Landrace | CRB-PT |  | NA |  |  |  |  |  |  |
| G80 | Martinique | Caribbean | Landrace | CRB-PT |  | NA |  |  |  |  |  |  |
| CRB48 | Martinique | Caribbean | Landrace | CRB-PT |  | 3 |  |  |  |  |  |  |
| CRB39 | Martinique | Caribbean | Landrace | CRB-PT |  | NA |  |  |  |  |  |  |
| G412 | Guadeloupe | Caribbean | Landrace | CRB-PT |  | 3 |  |  |  |  |  |  |
| G416 | Guadeloupe | Caribbean | Landrace | CRB-PT |  | 3 |  |  |  |  |  |  |
| CRB762 | Guadeloupe | Caribbean | Landrace | CRB-PT |  | 4 |  |  |  |  |  |  |
| G57 | Guadeloupe | Caribbean | Landrace | CRB-PT |  | NA |  |  |  |  |  |  |
| CRB73 | Guadeloupe | Caribbean | Landrace | CRB-PT |  | 2 | MLL12 |  |  |  |  |  |
| B25 | Guadeloupe | Caribbean | Siblings | Cirad | 2 | 2 |  |  |  |  |  |  |
| B21 | Guadeloupe | Caribbean | Siblings | Cirad | 2 | 2 |  |  |  |  |  |  |
| A57 | Guadeloupe | Caribbean | Siblings | Cirad |  | 2 |  |  |  |  |  |  |
| CRB88 | Guadeloupe | Caribbean | Landrace | CRB-PT |  | 3 |  |  |  |  |  |  |
| B154 | Guadeloupe | Caribbean | Siblings | Cirad |  | 2 |  |  |  |  |  |  |
| G455 | Guadeloupe | Caribbean | Hybrid | CRB-PT |  | 2 |  |  |  |  |  |  |
| G453 | Guadeloupe | Caribbean | Replicate | CRB-PT |  | 2 |  |  |  |  |  |  |
| A93 | Guadeloupe | Caribbean | Siblings | Cirad |  | 2 |  |  |  |  |  |  |
| B70 | Guadeloupe | Caribbean | Siblings | Cirad |  | 2 |  |  |  |  |  |  |
| KABUSA-Mbis | Guadeloupe | Caribbean | Landrace | Cirad |  | NA |  |  |  |  |  |  |
| G628 | Guadeloupe | Caribbean | Landrace | CRB-PT |  | 2 | MLL12 |  |  |  |  |  |
| B14 | Guadeloupe | Caribbean | Siblings | Cirad | 2 | 2 |  |  |  |  |  |  |
| CRB453 | Guadeloupe | Caribbean | Replicate | CRB-PT |  | 2 |  |  |  |  |  |  |
| G43 | Guadeloupe | Caribbean | Landrace | CRB-PT |  | NA |  |  |  |  |  |  |
| B31 | Guadeloupe | Caribbean | Siblings | Cirad |  | 2 |  |  |  |  |  |  |
| Roujol45 | Guadeloupe | Caribbean | Landrace | Cirad |  | 4 |  |  |  |  |  |  |
| A49 | Guadeloupe | Caribbean | Siblings | Cirad | 2 | 2 |  |  |  |  |  |  |
| Roujol39 | Guadeloupe | Caribbean | Landrace | Cirad |  | 2 | UG | UG | 0.00001 | 0.495372 | 0.128659 | 0.375959 |
| Roujol37 | Guadeloupe | Caribbean | Landrace | Cirad |  | 2 | UG | UG | 0.00001 | 0.99997 | 0.00001 | 0.00001 |
| CRB630 | Guadeloupe | Caribbean | Landrace | CRB-PT |  | 2 | MLL12 |  |  |  |  |  |
| G619 | Guadeloupe | Caribbean | Landrace | CRB-PT |  | NA |  |  |  |  |  |  |
| G614 | Guadeloupe | Caribbean | Landrace | CRB-PT |  | NA |  |  |  |  |  |  |
| CRB387 | Guadeloupe | Caribbean | Landrace | CRB-PT |  | 2 | MLL6 |  |  |  |  |  |
| CRB388 | Guadeloupe | Caribbean | Hybrid | CRB-PT |  | 2 |  |  |  |  |  |  |
| A80 | Guadeloupe | Caribbean | Siblings | Cirad |  | 2 |  |  |  |  |  |  |
| A88 | Guadeloupe | Caribbean | Siblings | Cirad |  | 2 |  |  |  |  |  |  |
| CRB416 | Guadeloupe | Caribbean | Landrace | CRB-PT |  | 2 | MLL7 |  |  |  |  |  |
| CRB417 | Guadeloupe | Caribbean | Hybrid | CRB-PT |  | 2 |  |  |  |  |  |  |
| CRB412 | Guadeloupe | Caribbean | Landrace | CRB-PT |  | 2 | MLL6 |  |  |  |  |  |
| CRB413 | Guadeloupe | Caribbean | Hybrid | CRB-PT |  | 2 |  |  |  |  |  |  |
| A76 | Guadeloupe | Caribbean | Siblings | Cirad |  | 2 |  |  |  |  |  |  |
| B108 | Guadeloupe | Caribbean | Siblings | Cirad |  | 2 |  |  |  |  |  |  |
| B191 | Guadeloupe | Caribbean | Siblings | Cirad |  | 2 |  |  |  |  |  |  |
| B193 | Guadeloupe | Caribbean | Siblings | Cirad |  | 2 |  |  |  |  |  |  |
| G372 | Guadeloupe | Caribbean | Landrace | CRB-PT |  | NA |  |  |  |  |  |  |
| CRB111 | Guadeloupe | Caribbean | Landrace | CRB-PT |  | 2 | MLL5 |  |  |  |  |  |
| CRB113 | Guadeloupe | Caribbean | Landrace | CRB-PT |  | NA |  |  |  |  |  |  |
| G70 | Guadeloupe | Caribbean | Landrace | CRB-PT |  | NA |  |  |  |  |  |  |
| G73 | Guadeloupe | Caribbean | Landrace | CRB-PT |  | NA |  |  |  |  |  |  |
| KABUSA-F | Guadeloupe | Caribbean | Landrace | Cirad |  | NA |  |  |  |  |  |  |
| A25 | Guadeloupe | Caribbean | Siblings | Cirad |  | 2 |  |  |  |  |  |  |
| A28 | Guadeloupe | Caribbean | Siblings | Cirad |  | 2 |  |  |  |  |  |  |
| B172 | Guadeloupe | Caribbean | Siblings | Cirad |  | 2 |  |  |  |  |  |  |
| CRB629 | Guadeloupe | Caribbean | Landrace | CRB-PT |  | 2 | MLL10 |  |  |  |  |  |
| CRB628 | Guadeloupe | Caribbean | Landrace | CRB-PT |  | 2 | MLL12 |  |  |  |  |  |
| G39 | Guadeloupe | Caribbean | Landrace | CRB-PT |  | 4 |  |  |  |  |  |  |
| KABUSA-Fbis | Guadeloupe | Caribbean | Landrace | Cirad |  | 3 |  |  |  |  |  |  |
| CRB473 | Guadeloupe | Caribbean | Landrace | CRB-PT |  | NA |  |  |  |  |  |  |
| CRB402 | Guadeloupe | Caribbean | Landrace | CRB-PT |  | 2 | MLL8 |  |  |  |  |  |
| CRB401 | Guadeloupe | Caribbean | Hybrid | CRB-PT |  | 2 |  |  |  |  |  |  |
| Roujol66 | Guadeloupe | Caribbean | Landrace | Cirad | 4 | 4 |  |  |  |  |  |  |
| Roujol65 | Guadeloupe | Caribbean | Landrace | Cirad | 4 | 4 |  |  |  |  |  |  |
| A69 | Guadeloupe | Caribbean | Siblings | Cirad |  | 2 |  |  |  |  |  |  |
| A67 | Guadeloupe | Caribbean | Siblings | Cirad |  | 2 |  |  |  |  |  |  |
| B9 | Guadeloupe | Caribbean | Siblings | Cirad | 2 | 2 |  |  |  |  |  |  |
| A115 | Guadeloupe | Caribbean | Siblings | Cirad | 2 | 2 |  |  |  |  |  |  |
| A81 | Guadeloupe | Caribbean | Siblings | Cirad |  | 2 |  |  |  |  |  |  |
| A87 | Guadeloupe | Caribbean | Siblings | Cirad |  | 2 |  |  |  |  |  |  |
| B214 | Guadeloupe | Caribbean | Siblings | Cirad |  | 2 |  |  |  |  |  |  |
| CRB759 | Guadeloupe | Caribbean | Landrace | CRB-PT |  | 3 |  |  |  |  |  |  |
| CRB617 | Guadeloupe | Caribbean | Landrace | CRB-PT | 2 | 2 | MLL4 |  |  |  |  |  |
| CRB753 | Guadeloupe | Caribbean | Landrace | CRB-PT |  | NA |  |  |  |  |  |  |
| CRB752 | Guadeloupe | Caribbean | Landrace | CRB-PT |  | 2 | MLL13 |  |  |  |  |  |
| CRB751 | Guadeloupe | Caribbean | Hybrid | CRB-PT |  | 2 |  |  |  |  |  |  |
| CRB750 | Guadeloupe | Caribbean | Landrace | CRB-PT |  | 3 |  |  |  |  |  |  |
| CRB755 | Guadeloupe | Caribbean | Landrace | CRB-PT |  | 2 | MLL6 |  |  |  |  |  |
| B10 | Guadeloupe | Caribbean | Siblings | Cirad |  | 2 |  |  |  |  |  |  |
| B18 | Guadeloupe | Caribbean | Siblings | Cirad | 2 | 2 |  |  |  |  |  |  |
| A10 | Guadeloupe | Caribbean | Siblings | Cirad | 2 | 2 |  |  |  |  |  |  |
| A2 | Guadeloupe | Caribbean | Siblings | Cirad |  | 2 |  |  |  |  |  |  |
| B161 | Guadeloupe | Caribbean | Siblings | Cirad |  | 2 |  |  |  |  |  |  |
| G630 | Guadeloupe | Caribbean | Landrace | CRB-PT |  | 2 | MLL37 | MLL37 | 0.00001 | 0.143052 | 0.00001 | 0.856928 |
| CRB76 | Puerto Rico | Caribbean | Landrace | CRB-PT | 2 | 2 | UG | UG | 0.00001 | 0.00001 | 0.00001 | 0.99997 |
| G59 | Puerto Rico | Caribbean | Landrace | CRB-PT |  | 2 | MLL9 |  |  |  |  |  |
| CRB96 | Puerto Rico | Caribbean | Landrace | CRB-PT |  | 2 | UG | UG | 0.057624 | 0.420592 | 0.355926 | 0.165857 |
| CRB22 | Puerto Rico | Caribbean | Landrace | CRB-PT |  | 2 | MLL11 |  |  |  |  |  |
| CRB454 | Puerto Rico | Caribbean | Landrace | CRB-PT |  | NA |  |  |  |  |  |  |
| CRB102 | Puerto Rico | Caribbean | Landrace | CRB-PT |  | NA |  |  |  |  |  |  |
| CRB68 | Puerto Rico | Caribbean | Landrace | CRB-PT |  | 2 | MLL13 |  |  |  |  |  |
| G41 | Puerto Rico | Caribbean | Landrace | CRB-PT |  | 2 | MLL4 |  |  |  |  |  |
| CRB61 | Puerto Rico | Caribbean | Landrace | CRB-PT |  | 2 | MLL12 |  |  |  |  |  |
| CRB65 | Puerto Rico | Caribbean | Landrace | CRB-PT | 2 | 2 | MLL13 |  |  |  |  |  |
| Roujol40 | Puerto Rico | Caribbean | Landrace | Cirad |  | 2 | UG | UG | 0.00001 | 0.652885 | 0.347095 | 0.00001 |
| CRB51 | Puerto Rico | Caribbean | Landrace | CRB-PT |  | 2 | UG | UG | 0.00001 | 0.419672 | 0.00001 | 0.580308 |
| CRB59 | Puerto Rico | Caribbean | Landrace | CRB-PT |  | 2 | MLL9 |  |  |  |  |  |
| CRB112 | Puerto Rico | Caribbean | Landrace | CRB-PT |  | 2 | UG | UG | 0.777953 | 0.05632 | 0.093127 | 0.072601 |
| CRB40 | Puerto Rico | Caribbean | Landrace | CRB-PT | 3 | 3 |  |  |  |  |  |  |
| CRB41 | Puerto Rico | Caribbean | Landrace | CRB-PT |  | 2 | MLL4 |  |  |  |  |  |
| G68 | Puerto Rico | Caribbean | Landrace | CRB-PT |  | 2 | MLL13 |  |  |  |  |  |
| G65 | Puerto Rico | Caribbean | Landrace | CRB-PT |  | 2 | MLL13 |  |  |  |  |  |
| CRB34 | Saint Lucia | Caribbean | Landrace | CRB-PT |  | 2 | MLL9 |  |  |  |  |  |
| CRB476 | Saint Vincent | Caribbean | Landrace | CRB-PT |  | 2 | UG | UG | 0.016542 | 0.096243 | 0.105404 | 0.781811 |
| CRB72 | New Caledonia | Pacific | Landrace | CRB-PT |  | 2 | MLL11 | MLL11 | 0.00001 | 0.08427 | 0.91571 | 0.00001 |
| CRB95 | New Caledonia | Pacific | Landrace | CRB-PT |  | 3 |  |  |  |  |  |  |
| CRB26 | New Caledonia | Pacific | Landrace | CRB-PT | 4 | 4 |  |  |  |  |  |  |
| G623 | New Caledonia | Pacific | Landrace | CRB-PT |  | 2 | MLL15 |  |  |  |  |  |
| CRB16 | New Caledonia | Pacific | Landrace | CRB-PT |  | NA |  |  |  |  |  |  |
| CRB83 | New Caledonia | Pacific | Landrace | CRB-PT |  | 3 |  |  |  |  |  |  |
| CRB53 | New Caledonia | Pacific | Landrace | CRB-PT |  | 2 | MLL10 |  |  |  |  |  |
| CRB623 | New Caledonia | Pacific | Landrace | CRB-PT |  | 2 | MLL12 |  |  |  |  |  |
| CRB622 | New Caledonia | Pacific | Landrace | CRB-PT |  | 4 |  |  |  |  |  |  |
| G66 | New Caledonia | Pacific | Landrace | CRB-PT |  | NA |  |  |  |  |  |  |
| CRB37 | New Caledonia | Pacific | Landrace | CRB-PT |  | NA |  |  |  |  |  |  |
| NARI-24 | Papua New Guinea | Pacific | Landrace | NARI |  | NA |  |  |  |  |  |  |
| NARI-17 | Papua New Guinea | Pacific | Landrace | NARI |  | NA |  |  |  |  |  |  |
| NARI-10 | Papua New Guinea | Pacific | Landrace | NARI |  | 2 | UG | UG | 0.016 | 0.348925 | 0.635066 | 0.00001 |
| Vua1004 | Vanuatu | Pacific | Landrace | VARTC |  | 2 | MLL40 |  |  |  |  |  |
| CRB767 | Vanuatu | Pacific | Landrace | CRB-PT |  | 2 | MLL13 |  |  |  |  |  |
| CRB768 | Vanuatu | Pacific | Landrace | CRB-PT |  | 2 | UG | UG | 0.110941 | 0.625351 | 0.263698 | 0.00001 |
| Vua40 | Vanuatu | Pacific | Landrace | VARTC |  | 2 | MLL3 |  |  |  |  |  |
| Vua1168 | Vanuatu | Pacific | Landrace | VARTC |  | 4 |  |  |  |  |  |  |
| Roujol25 | Vanuatu | Pacific | Landrace | Cirad |  | 3 |  |  |  |  |  |  |
| Vua1598 | Vanuatu | Pacific | Landrace | VARTC |  | NA |  |  |  |  |  |  |
| Vua1593 | Vanuatu | Pacific | Landrace | VARTC |  | 3 |  |  |  |  |  |  |
| Vua1459 | Vanuatu | Pacific | Landrace | VARTC |  | NA |  |  |  |  |  |  |
| Vua1597 | Vanuatu | Pacific | Landrace | VARTC |  | 2 | MLL40 | MLL40 | 0.15082 | 0.031629 | 0.669766 | 0.147785 |
| Vua12 | Vanuatu | Pacific | Landrace | VARTC |  | 2 | UG | UG | 0.00001 | 0.835972 | 0.00001 | 0.164008 |
| Vua1688 | Vanuatu | Pacific | Landrace | VARTC |  | NA |  |  |  |  |  |  |
| Vua1076 | Vanuatu | Pacific | Landrace | VARTC |  | 3 |  |  |  |  |  |  |
| BGPI-27 | Vanuatu | Pacific | Landrace | Cirad | 4 | 4 |  |  |  |  |  |  |
| Vua24 | Vanuatu | Pacific | Landrace | VARTC |  | 4 |  |  |  |  |  |  |
| CRB763 | Vanuatu | Pacific | Landrace | CRB-PT |  | 2 | MLL13 |  |  |  |  |  |
| Vua20 | Vanuatu | Pacific | Landrace | VARTC |  | NA |  |  |  |  |  |  |
| Vua1679 | Vanuatu | Pacific | Landrace | VARTC |  | 2 | MLL41 |  |  |  |  |  |
| Vua606 | Vanuatu | Pacific | Landrace | VARTC |  | NA |  |  |  |  |  |  |
| Vua1316 | Vanuatu | Pacific | Landrace | VARTC |  | 2 | MLL21 |  |  |  |  |  |
| Vua457 | Vanuatu | Pacific | Landrace | VARTC |  | 2 | MLL4 |  |  |  |  |  |
| Vua1590 | Vanuatu | Pacific | Landrace | VARTC |  | 2 | MLL41 |  |  |  |  |  |
| BGPI-9 | Vanuatu | Pacific | Landrace | Cirad |  | NA |  |  |  |  |  |  |
| Vua1339 | Vanuatu | Pacific | Landrace | VARTC |  | 2 | MLL41 |  |  |  |  |  |
| Vua725 | Vanuatu | Pacific | Landrace | VARTC |  | NA |  |  |  |  |  |  |
| Vua1538 | Vanuatu | Pacific | Landrace | VARTC |  | 2 | MLL41 |  |  |  |  |  |
| Vua720 | Vanuatu | Pacific | Landrace | VARTC |  | NA |  |  |  |  |  |  |
| Vua655 | Vanuatu | Pacific | Landrace | VARTC |  | 2 | MLL41 |  |  |  |  |  |
| Vua555 | Vanuatu | Pacific | Landrace | VARTC |  | 3 |  |  |  |  |  |  |
| Vua1448 | Vanuatu | Pacific | Landrace | VARTC |  | 2 | MLL13 |  |  |  |  |  |
| Vua1286 | Vanuatu | Pacific | Landrace | VARTC |  | 2 | MLL21 |  |  |  |  |  |
| Vua428b | Vanuatu | Pacific | Landrace | VARTC |  | 2 | UG | UG | 0.00001 | 0.00001 | 0.99997 | 0.00001 |
| BGPI-8 | Vanuatu | Pacific | Landrace | Cirad | 4 | 4 |  |  |  |  |  |  |
| Vua28 | Vanuatu | Pacific | Landrace | VARTC |  | NA |  |  |  |  |  |  |
| BGPI-10 | Vanuatu | Pacific | Landrace | Cirad |  | 2 | MLL3 | MLL3 | 0.00001 | 0.097221 | 0.902759 | 0.00001 |
| Vua03a | Vanuatu | Pacific | Landrace | VARTC |  | 3 |  |  |  |  |  |  |
| Vua729 | Vanuatu | Pacific | Landrace | VARTC |  | NA |  |  |  |  |  |  |
| Vua18a | Vanuatu | Pacific | Landrace | VARTC |  | 2 | MLL21 |  |  |  |  |  |
| Roujol31 | Vanuatu | Pacific | Landrace | Cirad |  | 3 |  |  |  |  |  |  |
| Vua1541 | Vanuatu | Pacific | Landrace | VARTC |  | 3 |  |  |  |  |  |  |
| Vua15 | Vanuatu | Pacific | Landrace | VARTC |  | NA |  |  |  |  |  |  |
| Vua32a | Vanuatu | Pacific | Landrace | VARTC |  | 2 | UG | UG | 0.00001 | 0.00001 | 0.99997 | 0.00001 |
| Vua1539 | Vanuatu | Pacific | Landrace | VARTC |  | 2 | MLL9 |  |  |  |  |  |
| Vua25 | Vanuatu | Pacific | Landrace | VARTC |  | NA |  |  |  |  |  |  |
| Vua264 | Vanuatu | Pacific | Landrace | VARTC |  | 2 | MLL42 |  |  |  |  |  |
| Vua1989 | Vanuatu | Pacific | Landrace | VARTC |  | 2 | MLL21 |  |  |  |  |  |
| Vua505 | Vanuatu | Pacific | Landrace | VARTC |  | 2 | MLL41 | MLL41 | 0.040311 | 0.00001 | 0.959669 | 0.00001 |
| Vua503 | Vanuatu | Pacific | Landrace | VARTC |  | 3 |  |  |  |  |  |  |
| Vua461 | Vanuatu | Pacific | Landrace | VARTC |  | 2 | MLL13 |  |  |  |  |  |
| Vua660 | Vanuatu | Pacific | Landrace | VARTC |  | NA |  |  |  |  |  |  |
| Vua482 | Vanuatu | Pacific | Landrace | VARTC |  | 2 | MLL41 |  |  |  |  |  |
| Vua1029 | Vanuatu | Pacific | Landrace | VARTC |  | NA |  |  |  |  |  |  |
| Vua30a | Vanuatu | Pacific | Landrace | VARTC |  | 2 | MLL41 |  |  |  |  |  |
| Vua1419 | Vanuatu | Pacific | Landrace | VARTC |  | NA |  |  |  |  |  |  |
| Roujol72 | Vanuatu | Pacific | Landrace | Cirad |  | 2 | MLL13 |  |  |  |  |  |
| Vua717 | Vanuatu | Pacific | Landrace | VARTC |  | 2 | MLL41 |  |  |  |  |  |
| Vua1006 | Vanuatu | Pacific | Landrace | VARTC |  | NA |  |  |  |  |  |  |
| Vua1618 | Vanuatu | Pacific | Landrace | VARTC |  | 3 |  |  |  |  |  |  |
| Vua549 | Vanuatu | Pacific | Landrace | VARTC |  | NA |  |  |  |  |  |  |
| Vua10 | Vanuatu | Pacific | Hybrid | VARTC |  | NA |  |  |  |  |  |  |
| Vua280 | Vanuatu | Pacific | Landrace | VARTC |  | 2 | UG | UG | 0.00001 | 0.00001 | 0.99997 | 0.00001 |
| Vua38 | Vanuatu | Pacific | Landrace | VARTC |  | NA |  |  |  |  |  |  |
| Vua34 | Vanuatu | Pacific | Hybrid | VARTC |  | 2 |  |  |  |  |  |  |
| Vua35 | Vanuatu | Pacific | Landrace | VARTC |  | 2 | MLL4 |  |  |  |  |  |
| Vua36 | Vanuatu | Pacific | Landrace | VARTC |  | 2 | MLL13 |  |  |  |  |  |
| Vua37 | Vanuatu | Pacific | Landrace | VARTC |  | 2 | MLL13 | MLL13 | 0.00001 | 0.151799 | 0.848181 | 0.00001 |
| Vua756 | Vanuatu | Pacific | Landrace | VARTC |  | NA |  |  |  |  |  |  |
| Vua754 | Vanuatu | Pacific | Landrace | VARTC |  | NA |  |  |  |  |  |  |
| Roujol29 | Vanuatu | Pacific | Landrace | Cirad | 4 | 4 |  |  |  |  |  |  |
| Vua49 | Vanuatu | Pacific | Landrace | VARTC |  | 2 | MLL42 | MLL42 | 0.006207 | 0.00001 | 0.993773 | 0.00001 |
| Roujol23 | Vanuatu | Pacific | Landrace | Cirad |  | 3 |  |  |  |  |  |  |
| Roujol21 | Vanuatu | Pacific | Landrace | Cirad | 4 | 4 |  |  |  |  |  |  |
| Roujol27 | Vanuatu | Pacific | Landrace | Cirad | 4 | 4 |  |  |  |  |  |  |
| Roujol26 | Vanuatu | Pacific | Landrace | Cirad |  | 2 | MLL4 |  |  |  |  |  |
| Vua432 | Vanuatu | Pacific | Landrace | VARTC |  | 2 | MLL21 |  |  |  |  |  |
| Vua1491 | Vanuatu | Pacific | Landrace | VARTC |  | NA |  |  |  |  |  |  |
| Vua1445 | Vanuatu | Pacific | Landrace | VARTC |  | NA |  |  |  |  |  |  |
| Vua1486 | Vanuatu | Pacific | Landrace | VARTC |  | 2 | MLL21 |  |  |  |  |  |
| Vua536 | Vanuatu | Pacific | Landrace | VARTC |  | 3 |  |  |  |  |  |  |
| Vua1481 | Vanuatu | Pacific | Landrace | VARTC |  | 2 | MLL13 |  |  |  |  |  |
| Vua1046 | Vanuatu | Pacific | Landrace | VARTC |  | 2 | MLL42 |  |  |  |  |  |
| Vua474 | Vanuatu | Pacific | Landrace | VARTC |  | 2 | MLL41 |  |  |  |  |  |
| Vua672 | Vanuatu | Pacific | Landrace | VARTC |  | 2 | MLL41 |  |  |  |  |  |
| Vua497 | Vanuatu | Pacific | Landrace | VARTC |  | 2 | MLL4 |  |  |  |  |  |
| Vua491 | Vanuatu | Pacific | Landrace | VARTC |  | 2 | MLL21 |  |  |  |  |  |
| Vua408 | Vanuatu | Pacific | Landrace | VARTC |  | 2 | MLL15 | MLL15 | 0.00001 | 0.00001 | 0.99997 | 0.00001 |
| Vua402 | Vanuatu | Pacific | Landrace | VARTC |  | 2 | MLL13 |  |  |  |  |  |
| Vua03 | Vanuatu | Pacific | Landrace | VARTC |  | NA |  |  |  |  |  |  |
| Vua07 | Vanuatu | Pacific | Hybrid | VARTC |  | NA |  |  |  |  |  |  |
| Vua08 | Vanuatu | Pacific | Hybrid | VARTC |  | NA |  |  |  |  |  |  |
| Roujol1 | Vanuatu | Pacific | Landrace | Cirad |  | 3 |  |  |  |  |  |  |
| Roujol4 | Vanuatu | Pacific | Landrace | Cirad |  | 3 |  |  |  |  |  |  |
| Roujol5 | Vanuatu | Pacific | Landrace | Cirad |  | 4 |  |  |  |  |  |  |
| Vua533 | Vanuatu | Pacific | Landrace | VARTC |  | 2 | MLL3 |  |  |  |  |  |
| Roujol2 | Vanuatu | Pacific | Landrace | Cirad |  | 2 | UG | UG | 0.00001 | 0.756408 | 0.243572 | 0.00001 |
| Roujol3 | Vanuatu | Pacific | Landrace | Cirad | 4 | 4 |  |  |  |  |  |  |
| Roujol8 | Vanuatu | Pacific | Landrace | Cirad |  | 3 |  |  |  |  |  |  |
| Roujol9 | Vanuatu | Pacific | Landrace | Cirad | 4 | 4 |  |  |  |  |  |  |
| Vua20a | Vanuatu | Pacific | Landrace | VARTC |  | NA |  |  |  |  |  |  |
| Vua1442 | Vanuatu | Pacific | Landrace | VARTC |  | NA |  |  |  |  |  |  |
| Vua448 | Vanuatu | Pacific | Landrace | VARTC |  | NA |  |  |  |  |  |  |
| Vua567 | Vanuatu | Pacific | Landrace | VARTC |  | NA |  |  |  |  |  |  |
| Vua522 | Vanuatu | Pacific | Landrace | VARTC |  | NA |  |  |  |  |  |  |
| Roujol17 | Vanuatu | Pacific | Landrace | Cirad |  | 2 | MLL40 |  |  |  |  |  |
| Roujol11 | Vanuatu | Pacific | Landrace | Cirad | 2 | 2 | UG | UG | 0.00001 | 0.26284 | 0.722198 | 0.014952 |
| Vua1485 | Vanuatu | Pacific | Landrace | VARTC |  | NA |  |  |  |  |  |  |
| Vua1480 | Vanuatu | Pacific | Landrace | VARTC |  | NA |  |  |  |  |  |  |
| Vua1483 | Vanuatu | Pacific | Landrace | VARTC |  | 2 | MLL42 |  |  |  |  |  |
| Vua39 | Vanuatu | Pacific | Landrace | VARTC |  | 2 | UG | UG | 0.00001 | 0.00001 | 0.99997 | 0.00001 |

**Table S3:** Geographical distribution of diploid genotypes showing membership in each of the four clusters (*K=4*) and admixed groups identified after analysis of the population structure using ADMIXTURE.

| ***K=4*** | Cluster_1 | Cluster_2 | Cluster_3 | Cluster_4 | Admixed | Total |
| --- | --- | --- | --- | --- | --- | --- |
| Africa |  | 1 | 1 | 8 | 6 | 16 |
| Mainland Southeast Asia | 18 |  | 1 |  | 9 | 28 |
| Indian Peninsula |  | 2 |  | 1 | 7 | 10 |
| Caribbean | 1 | 4 |  | 3 | 15 | 23 |
| Pacific |  | 1 | 10 |  | 5 | 16 |


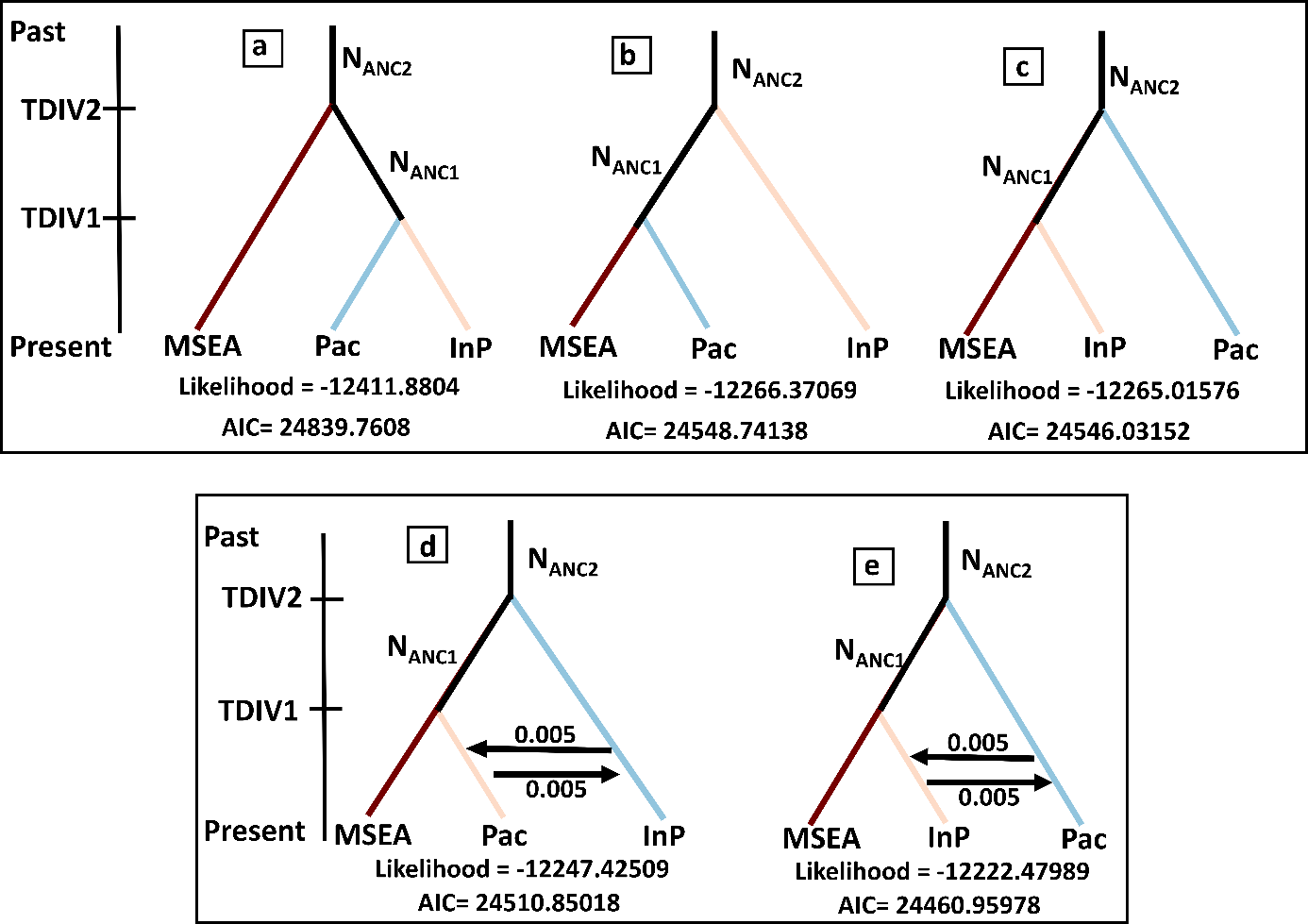


**Fig. S7**. The different domestication scenarios simulated with FASTSIMCOAL 2.6*.*

Scenarios (a, b, c): (a) Split between Mainland Southeast Asia (MSEA) and the Indian Peninsula (InP) followed by the divergence of a Pacific (Pac) population from InP or (b) MSEA. (c) Split between MSEA and Pacific populations, with Indian Peninsula populations originating from MSEA. Scenarios (d, e): (d) and (e) are equivalent to the scenarios (b) and (c), including constant migration between MSEA and the Pacific and between the Indian Peninsula and the Pacific, respectively.

Nanc1 ancestral population after the first split. TDIV: Time of divergence between the populations; TDIV1 is the most recent time of divergence. TDI1<TIV2. 0.05 is the migration rate between two populations.

**Detailed method description S1:** PolynomPloidy.R Script

**PolynomPloidy.R**

#Copyright 2020 CIRAD

#

# This program is free software; you can redistribute it and/or modify

# it under the terms of the GNU General Public License as published by

# the Free Software Foundation; either version 3 of the License, or

# (at your option) any later version.

#

# This program is distributed in the hope that it will be useful,

# but WITHOUT ANY WARRANTY; without even the implied warranty of

# MERCHANTABILITY or FITNESS FOR A PARTICULAR PURPOSE. See the

# GNU General Public License for more details.

#

# You should have received a copy of the GNU General Public License

# along with this program; if not, see <http://www.gnu.org/licenses/> or

# write to the Free Software Foundation, Inc.,

# 51 Franklin Street, Fifth Floor, Boston,

# MA 02110-1301, USA.

#

# You should have received a copy of the CeCILL-C license with this program.

#If not see <http://www.cecill.info/licences/Licence_CeCILL-C_V1-en.txt>

#

# Intellectual property belongs to CIRAD

#

# Written by Fabien Cormier

####################################################################

#The PolynomPloidy.R estimates ploidy level from GBS dataset.

#A VCF file is taken as an input. For model training, information on accessions with known ploidy are provided as a text file #with two columns: accessions IDs are in the first column and the associated ploidy level in the second one. #PolynomPloidy.R #generates two main files: a text file with the ploidy level of each accession, present in the VCF file along #with a fitness value; #and a pdf file with the frequency distribution plot for each accession. Poor fit lead to the report of a ploidy levels of 1.

######################################################################
**library**(vcfR)
**library**(stringr)

######################################################################
*# 0 - IMPORTING THE VCF AND PREPARING THE MATRICES*
######################################################################
*#working directory*
**setwd**("Path to working directory")
*#* *Read the ploidy of training accessions*
var<-**read.table**("input_file_for_training.txt",sep="\t",row.names=1,header=T)
ok<-**row.names**(var)
*#read the vcf*
GBS<-**read.vcfR**("input_file.vcf")
*#* *Extract GBS info*
Z<-**extract_gt_tidy**(GBS)
*#* *transform depth info into 2 columns (1 per alleles)*
AD<-**str_split_fixed**(Z**$**gt_AD,",",2)
*#* *Put NA if there are 2 or more alternative alleles*
AD[**grep**(",",AD[,2]),]<-NA
*#* *Keep the depth of the major allele*
MAX_AD<-**apply**(AD,1,FUN=**function**(x){**max**(**as.numeric**(x),na.rm=T)})
MAX_AD[MAX_AD**==**"-Inf"]<-NA
MAX_AD<-**data.frame**(AD,MAX_AD,Z**$**gt_DP,Z**$**Indiv,Z**$**Key)
*#* *Create a total depth matrix by ind and SNP*
DP1 <- **extract.gt**(GBS, element = "DP", as.numeric=TRUE)
*#* *Create a major allele depth matrix by ind and SNP*
MAX1<-**matrix**(MAX_AD**$**MAX,ncol=**ncol**(DP1),nrow=**nrow**(DP1),byrow=F)
**colnames**(MAX1)<-**colnames**(DP1)
**row.names**(MAX1)<-**row.names**(DP1)

######################################################################
*# 1 - TEST the PARAMETRES*

######################################################################
*#* *Parameters (start, end, step)*

*#max number of reads per snp*
**hist**(**rowSums**(DP1,na.rm=T))
**summary**(**rowSums**(DP1,na.rm=T))
tmaxDPtot<-**seq**(75000,150000,25000)
*#min and max number of reads per SNP and ind*
**hist**(**as.vector**(DP1),xlim=**c**(0,1000),breaks=1000)
**summary**(**as.vector**(DP1),na.rm=T)
tminDP<-**seq**(10,50,10)
tmaxDP<-**seq**(200,800,200)
*#* *max frequency to take into account*
**hist**(**as.vector**(MAX1**/**DP1),breaks=100)
**summary**(**as.vector**(MAX1**/**DP1))
tfreqMaj<-**seq**(0.85,1,0.05)
*#* *number of histograms breaks*
tNbBreaks<-**c**(100,250,500)

*#* *Compute all combinations of parameters*
test<-**expand.grid**(tmaxDPtot,tminDP,tmaxDP,tfreqMaj,tNbBreaks)
**colnames**(test)<-**c**("maxDPtot","minDP","maxDP","freqMaj","NbBreaks")
*# Initialize variables*

nb_pred<-**as.numeric**();nb_ok<-**as.numeric**();nb_ploidy<-**as.numeric**()
mean_r2<-**as.numeric**()

*# loop on parameters combinations*
**for** (k **in** 1**:nrow**(test))
{
 **print**(**paste**("Test ====> ",**round**(k**/nrow**(test)*****100,2),"%","done",sep=" "))
 maxDPtot<-test[k,1];minDP<-test[k,2];maxDP<-test[k,3]
 freqMaj<-test[k,4];NbBreaks<-test[k,5]
 *#DP total max*
 MAX<-MAX1[**-which**(**rowSums**(DP1,na.rm=T)**>=**maxDPtot),]
 DP<-DP1[**-which**(**rowSums**(DP1,na.rm=T)**>=**maxDPtot),]
 *#NA if DP<=minDP or >=maxDP*
 MAX[DP**<=**minDP]<-NA
 MAX[DP**>=**maxDP]<-NA
 DP[**is.na**(MAX)**==**T]<-NA
 *#* *NA if major allele freq >= freqMaj*
 MAX[MAX**/**DP**>=**freqMaj]<-NA
 DP[**is.na**(MAX)**==**T]<-NA
 var_test<-**intersect**(**row.names**(var),**colnames**(MAX))
 MAX<-MAX[,var_test];DP<-DP[,var_test]
 ploidy<-**as.numeric**();fit<-**as.numeric**()

*# loop on accessions used as training dataset*
 **for** (i **in** 1**:ncol**(DP))
 {
 d<-**c**(MAX[,i]**/**DP[,i],(DP[,i]**-**MAX[,i])**/**DP[,i])
 **if**(**length**(**which**(**is.na**(d)**==**F))**<=**100)
 {ploidy<-**c**(ploidy,NA);fit<-**c**(fit,NA);**next**}
 h<-**hist**(d,breaks=NbBreaks,plot=F)
 y<-h**$**counts
 x<-**seq**((h**$**breaks[2]**-**h**$**breaks[1])**/**2**+**h**$**breaks[1],**max**(h**$**breaks),h**$**breaks[2]**-**h**$**breaks[1])
 modele<-**lm**(y**~poly**(x,8,raw=T))
 x<-**seq**(0.2,0.8,by=0.001)
 pred<-**predict**(modele,**data.frame**(x))
 nb_pic<-0
 **for** (j **in** 2**:**(**length**(pred)**-**1))
 {
 **if**(pred[j**-**1]**<=**pred[j] **&** pred[j]**>=**pred[j**+**1]){nb_pic<-nb_pic**+**1}
 }
 ploidy<-**c**(ploidy,nb_pic**+**1);fit<-**c**(fit,**summary**(modele)**$**"adj.r.squared")
 }
 nb_pred<-**c**(nb_pred,**length**(**which**(**is.na**(ploidy)**==**F)))
 nb_ok<-**c**(nb_ok,**length**(**which**(ploidy**==**var[var_test,1])))
 nb_ploidy<-**c**(nb_ploidy,**nlevels**(**as.factor**(ploidy[**which**(ploidy**==**var[var_test,1])])))
 mean_r2<-**c**(mean_r2,**mean**(fit,na.rm=T))
}
perc_ok<-nb_ok**/**nb_pred
RESULT_TEST<-**data.frame**(test,nb_pred,nb_ok,nb_ploidy,perc_ok,mean_r2)
*#* *or sort on the number of well predicted*
RESULT_TEST<-RESULT_TEST[**order**(RESULT_TEST**$**mean_r2,decreasing=T),]
RESULT_TEST<-RESULT_TEST[**order**(RESULT_TEST**$**nb_ploidy,decreasing=T),]
RESULT_TEST<-RESULT_TEST[**order**(RESULT_TEST**$**nb_ok,decreasing=T),]
RESULT_TEST[1**:**5,]
**write.table**(RESULT_TEST,file="Results_training.txt",sep="\t",dec=".",row.names=F)

######################################################################
*#2 - MAKE THE PREDICTION FOR ALL*
######################################################################
*#* *Parameters IF YOU DID NOT TRAIN THE MODEL*

*#number read max for 1 snp*
maxDPtot<-100000
*#* *number read min and max by 1 SNP and 1 genotype*

minDP<-30
maxDP<-600
*#* *frequency max to take into account as heterozygote*
freqMaj<-0.95
*#* *number breaks histogram*
NbBreaks<-250

*#* *OR CHOOSE THE MOST SUITABLE PARAMETERS USING TRAINING RESULTS*
RESULT_TEST[1,]
maxDPtot<-RESULT_TEST[1,1];minDP<-RESULT_TEST[1,2];maxDP<-RESULT_TEST[1,3]
freqMaj<-RESULT_TEST[1,4];NbBreaks<-RESULT_TEST[1,5]
*#* *basic matrix*
MAX<-MAX1
DP<-DP1
*#DP total max*
**hist**(**rowSums**(DP,na.rm=T));**abline**(h=maxDPtot)
MAX<-MAX[**-which**(**rowSums**(DP,na.rm=T)**>=**maxDPtot),]
DP<-DP[**-which**(**rowSums**(DP,na.rm=T)**>=**maxDPtot),]
*#NA if DP<=minDP or >=maxDP*
**hist**(DP,xlim=**c**(0,1000));**abline**(h=**c**(minDP,maxDP))
MAX[DP**<=**minDP]<-NA
MAX[DP**>=**maxDP]<-NA
DP[**is.na**(MAX)**==**T]<-NA
*#* *Check distribution of freq maj allele*
**hist**(MAX**/**DP)
*#* *If the allele maj = total for 1 geno 1 snp no variation => NA*
MAX[MAX**/**DP**>=**freqMaj]<-NA
DP[**is.na**(MAX)**==**T]<-NA
*#* *Loop on individuals*
**pdf**(**paste**("Results_ploidy_capt",maxDPtot,"minDP",minDP,"maxDP",maxDP,
 "freqMaj",freqMaj,"NbBreaks",NbBreaks,".pdf",sep=""),width = 21, height = 15)
nb_snp<-**apply**(MAX,2,FUN=**function**(x){**length**(**which**(**is.na**(x)**==**F))})
mean_DP<-**apply**(MAX,2,FUN=mean,na.rm=T)
ploidy<-**as.numeric**();fit<-**as.numeric**()
**for** (i **in** 1**:ncol**(DP))
{
 **print**(**paste**(**colnames**(DP)[i],"=",**round**(i**/dim**(DP)[2]*****100,2),"%","done",sep=" "))
 d<-**c**(MAX[,i]**/**DP[,i],(DP[,i]**-**MAX[,i])**/**DP[,i])
 **if**(**length**(**which**(**is.na**(d)**==**F))**<=**5)
 {ploidy<-**c**(ploidy,NA);fit<-**c**(fit,NA);**next**}
 h<-**hist**(d,breaks=NbBreaks,plot=F)
 **plot**(h,main=**colnames**(DP)[i])
 y<-h**$**counts
 x<-**seq**((h**$**breaks[2]**-**h**$**breaks[1])**/**2**+**h**$**breaks[1],**max**(h**$**breaks),h**$**breaks[2]**-**h**$**breaks[1])
 modele<-**lm**(y**~poly**(x,8,raw=T))
 x<-**seq**(0,1,by=0.01)
 **lines**(x,**predict**(modele,**data.frame**(x)),col="red")
 x<-**seq**(0.2,0.8,by=0.001)
 pred<-**predict**(modele,**data.frame**(x))
 nb_pic<-0
 **for** (j **in** 2**:**(**length**(pred)**-**1))
 {
 **if**(pred[j**-**1]**<=**pred[j] **&** pred[j]**>=**pred[j**+**1]){nb_pic<-nb_pic**+**1}
 }
 ploidy<-**c**(ploidy,nb_pic**+**1);fit<-**c**(fit,**summary**(modele)**$**"adj.r.squared")
}
**dev.off**()
RESULT_PLOIDY<-**data.frame**(ploidy,fit,nb_snp,mean_DP)
RESULT_PLOIDY<-**merge**(RESULT_PLOIDY,var,by="row.names",all.x=T)
RESULT_PLOIDY[**which**(RESULT_PLOIDY**$**ploidy**==**RESULT_PLOIDY**$**Ploidy),"OK"]<-"OK"
RESULT_PLOIDY[**which**(RESULT_PLOIDY**$**ploidy**!=**RESULT_PLOIDY**$**Ploidy),"OK"]<-"F"
RESULT_PLOIDY<-RESULT_PLOIDY[**order**(RESULT_PLOIDY**$**OK,RESULT_PLOIDY**$**Ploidy),]
**write.table(RESULTAT_PLOIDIE,file=paste("Results_ploidy",maxDPtot,**

**"minDP",minDP,"maxDP",maxDP,"freqMaj",freqMaj,**

**"NbBreaks",NbBreaks,".txt",sep=""),dec=".", sep="\t",row.names=F,quote=F)**

**Detailed method description S2:** Description of the demographic models tested.

Model_a.tpl

//Parameters for the coalescence simulation program:simcoal.exe

3

//Population effective sizes (number of genes)

NPOP0$

NPOP1$

NPOP2$

//Samples sizes and samples time and inbreeding

50

18

28

//Growth rates: negative growth implies population expansion

0

0

0

//Number of migration matrices:

0

//historical event: time, source, sink, migrants, new deme size, growth rate, migr mat index

2 historical event

TDIV1$ 2 1 1 RESIZE1$ 0 0

TDIV2$ 1 0 1 RESIZE2$ 0 0

//Number of independent loci [chromosome]

1 0

//Per chromosome: Number of contiguous linkage Block: a block is a set of contiguous loci

1

//per Block:data type, number of loci, per gen recomb and mut rates

FREQ 1 0 MUTATE OUTEXP

Model_a.est

// Priors and rules file

// *********************

[PARAMETERS]

//#isInt? #name #dist.#min #max

//all N are in number of haploid individuals

1 NPOP0$ logunif 100 100000 output

1 NPOP1$ logunif 100 100000 output

1 NPOP2$ logunif 100 100000 output

1 TDIV1$ logunif 50 20000 output

1 TDIV2$ logunif 50 20000 output

0 RESIZE1$ logunif 0.01 100 output

0 RESIZE2$ logunif 0.01 100 output

0 MUTATE unif 1e-7 1e-9 output

[RULES]

TDIV2$ > TDIV1$

[COMPLEX PARAMETERS]

1 ANCSIZE1$ = NPOP1$ * RESIZE1$ output

1 ANCSIZE2$ = NPOP0$ * RESIZE2$ output

Model_b.tpl

//Parameters for the coalescence simulation program:simcoal.exe

3

//Population effective sizes (number of genes)

NPOP0$

NPOP1$

NPOP2$

//Samples sizes and samples time and inbreeding

50

18

28

//Growth rates: negative growth implies population expansion

0

0

0

//Number of migration matrices :

0

//historical event: time, source, sink, migrants, new deme size, growth rate, migr mat index

2 historical event

TDIV1$ 2 0 1 RESIZE1$ 0 0

TDIV2$ 0 1 1 RESIZE2$ 0 0

//Number of independent loci [chromosome]

1 0

//Per chromosome: Number of contiguous linkage Block: a block is a set of contiguous loci

1

//per Block:data type, number of loci, per gen recomb and mut rates

FREQ 1 0 MUTATE OUTEXP

Model_b.est

// Priors and rules file

// *********************

[PARAMETERS]

//#isInt? #name #dist.#min #max

//all N are in number of haploid individuals

1 NPOP0$ logunif 100 100000 output

1 NPOP1$ logunif 100 100000 output

1 NPOP2$ logunif 100 100000 output

1 TDIV1$ logunif 50 20000 output

1 TDIV2$ logunif 50 20000 output

0 RESIZE1$ logunif 0.01 100 output

0 RESIZE2$ logunif 0.01 100 output

0 MUTATE unif 1e-7 1e-9 output

[RULES]

TDIV2$ > TDIV1$

[COMPLEX PARAMETERS]

1 ANCSIZE1$ = NPOP0$ * RESIZE1$ output

1 ANCSIZE2$ = NPOP1$ * RESIZE2$ output

Model_c.tpl

//Parameters for the coalescence simulation program:simcoal.exe

3

//Population effective sizes (number of genes)

NPOP0$

NPOP1$

NPOP2$

//Samples sizes and samples time and inbreeding

50

18

28

//Growth rates: negative growth implies population expansion

0

0

0

//Number of migration matrices :

0

//historical event: time, source, sink, migrants, new deme size, growth rate, migr mat index

2 historical event

TDIV1$ 1 0 1 RESIZE1$ 0 0

TDIV2$ 2 0 1 RESIZE2$ 0 0

//Number of independent loci [chromosome]

1 0

//Per chromosome: Number of contiguous linkage Block: a block is a set of contiguous loci

1

//per Block:data type, number of loci, per gen recomb and mut rates

FREQ 1 0 MUTATE OUTEXP

Model_c.est

// Priors and rules file

// *********************

[PARAMETERS]

//#isInt? #name #dist.#min #max

//all N are in number of haploid individuals

1 NPOP0$ logunif 100 100000 output

1 NPOP1$ logunif 100 100000 output

1 NPOP2$ logunif 100 100000 output

1 TDIV1$ logunif 50 20000 output

1 TDIV2$ logunif 50 20000 output

0 RESIZE1$ logunif 0.01 100 output

0 RESIZE2$ logunif 0.01 100 output

0 MUTATE unif 1e-7 1e-9 output

[RULES]

TDIV2$ > TDIV1$

[COMPLEX PARAMETERS]

1 ANCSIZE1$ = NPOP0$ * RESIZE1$ output

1 ANCSIZE2$ = NPOP0$ * RESIZE2$ output

Model_d.tpl

//Parameters for the coalescence simulation program:simcoal.exe

3

//Population effective sizes (number of genes)

NPOP0$

NPOP1$

NPOP2$

//Samples sizes and samples time and inbreeding

50

18

28

//Growth rates: negative growth implies population expansion

0

0

0

//Number of migration matrices :

2

//migration matrix 0

0 0 0

0 0 0.005

0 0.005 0

//migration matrix 1

0 0 0

0 0 0

0 0 0

//historical event: time, source, sink, migrants, new deme size, growth rate, migr mat index

2 historical event

TDIV1$ 2 0 1 RESIZE1$ 0 1

TDIV2$ 0 1 1 RESIZE2$ 0 1

//Number of independent loci [chromosome]

1 0

//Per chromosome: Number of contiguous linkage Block: a block is a set of contiguous loci

1

//per Block:data type, number of loci, per gen recomb and mut rates

FREQ 1 0 MUTATE OUTEXP

Model_d.est

// Priors and rules file

// *********************

[PARAMETERS]

//#isInt? #name #dist.#min #max

//all N are in number of haploid individuals

1 NPOP0$ logunif 100 100000 output

1 NPOP1$ logunif 100 100000 output

1 NPOP2$ logunif 100 100000 output

1 TDIV1$ logunif 50 20000 output

1 TDIV2$ logunif 50 20000 output

0 RESIZE1$ logunif 0.01 100 output

0 RESIZE2$ logunif 0.01 100 output

0 MUTATE unif 1e-7 1e-9 output

[RULES]

TDIV2$ > TDIV1$

[COMPLEX PARAMETERS]

1 ANCSIZE1$ = NPOP0$ * RESIZE1$ output

1 ANCSIZE2$ = NPOP1$ * RESIZE2$ output

Model_e.tpl

//Parameters for the coalescence simulation program:simcoal.exe

3

//Population effective sizes (number of genes)

NPOP0$

NPOP1$

NPOP2$

//Samples sizes and samples time and inbreeding

50

18

28

//Growth rates: negative growth implies population expansion

0

0

0

//Number of migration matrices :

2

//migration matrix 0

0 0 0

0 0 0.005

0 0.005 0

//migration matrix 1

0 0 0

0 0 0

0 0 0

//historical event: time, source, sink, migrants, new deme size, growth rate, migr mat index

2 historical event

TDIV1$ 1 0 1 RESIZE1$ 0 1

TDIV2$ 2 0 1 RESIZE2$ 0 1

//Number of independent loci [chromosome]

1 0

//Per chromosome: Number of contiguous linkage Block: a block is a set of contiguous loci

1

//per Block:data type, number of loci, per gen recomb and mut rates

FREQ 1 0 MUTATE OUTEXP

Model_e.est

// Priors and rules file

// *********************

[PARAMETERS]

//#isInt? #name #dist.#min #max

//all N are in number of haploid individuals

1 NPOP0$ logunif 100 100000 output

1 NPOP1$ logunif 100 100000 output

1 NPOP2$ logunif 100 100000 output

1 TDIV1$ logunif 50 20000 output

1 TDIV2$ logunif 50 20000 output

0 RESIZE1$ logunif 0.01 100 output

0 RESIZE2$ logunif 0.01 100 output

0 MUTATE unif 1e-7 1e-9 output

[RULES]

TDIV2$ > TDIV1$

[COMPLEX PARAMETERS]

1 ANCSIZE1$ = NPOP0$ * RESIZE1$ output

1 ANCSIZE2$ = NPOP0$ * RESIZE2$ output

Model_f.tpl

//Parameters for the coalescence simulation program:simcoal.exe

4

//Population effective sizes (number of genes)

NPOP0$

NPOP1$

NPOP2$

NPOP3$

//Samples sizes and samples time and inbreeding

28

50

18

28

//Growth rates: negative growth implies population expansion

0

0

0

0

//Number of migration matrices:

2

//migration matrix

0 0 0 0

0 0 0 0

0 0 0 0.005

0 0 0.005 0

//migration matrix

0 0 0 0

0 0 0 0

0 0 0 0

0 0 0 0

//historical event: time, source, sink, migrants, new deme size, growth rate, migr mat index

3 historical event

TDIV1$ 0 2 1 RESIZE1$ 0 0

TDIV2$ 1 2 1 RESIZE2$ 0 1

TDIV3$ 3 2 1 RESIZE3$ 0 1

//Number of independent loci [chromosome]

1 0

//Per chromosome: Number of contiguous linkage Block: a block is a set of contiguous loci

1

//per Block:data type, number of loci, per gen recomb and mut rates

FREQ 1 0 MUTATE OUTEXP

Model_f.est

// Priors and rules file

// *********************

[PARAMETERS]

//#isInt? #name #dist.#min #max

//all N are in number of haploid individuals

1 NPOP0$ logunif 100 100000 output

1 NPOP1$ logunif 100 100000 output

1 NPOP2$ logunif 100 100000 output

1 NPOP3$ logunif 100 100000 output

1 TDIV1$ logunif 50 20000 output

1 TDIV2$ logunif 50 20000 output

1 TDIV3$ logunif 50 20000 output

0 RESIZE1$ logunif 0.01 100 output

0 RESIZE2$ logunif 0.01 100 output

0 RESIZE3$ logunif 0.01 100 output

0 MUTATE unif 1e-7 1e-9 output

[RULES]

TDIV2$ > TDIV1$

TDIV3$ > TDIV2$

[COMPLEX PARAMETERS]

1 ANCSIZE1$ = NPOP2$ * RESIZE1$ output

1 ANCSIZE2$ = NPOP2$ * RESIZE2$ output

1 ANCSIZE3$ = NPOP2$ * RESIZE3$ output

Model_g.tpl

//Parameters for the coalescence simulation program:simcoal.exe

4

//Population effective sizes (number of genes)

NPOP0$

NPOP1$

NPOP2$

NPOP3$

//Samples sizes and samples time and inbreeding

28

50

18

28

//Growth rates: negative growth implies population expansion

0

0

0

0

//Number of migration matrices:

2

//migration matrix

0 0 0 0

0 0 0 0

0 0 0 0.005

0 0 0.005 0

//migration matrix

0 0 0 0

0 0 0 0

0 0 0 0

0 0 0 0

//historical event: time, source, sink, migrants, new deme size, growth rate, migr mat index

3 historical event

TDIV1$ 0 3 1 RESIZE1$ 0 0

TDIV2$ 1 2 1 RESIZE2$ 0 1

TDIV3$ 3 2 1 RESIZE3$ 0 1

//Number of independent loci [chromosome]

1 0

//Per chromosome: Number of contiguous linkage Block: a block is a set of contiguous loci

1

//per Block:data type, number of loci, per gen recomb and mut rates

FREQ 1 0 MUTATE OUTEXP

Model_g.est

// Priors and rules file

// *********************

[PARAMETERS]

//#isInt? #name #dist.#min #max

//all N are in number of haploid individuals

1 NPOP0$ logunif 100 100000 output

1 NPOP1$ logunif 100 100000 output

1 NPOP2$ logunif 100 100000 output

1 NPOP3$ logunif 100 100000 output

1 TDIV1$ logunif 50 20000 output

1 TDIV2$ logunif 50 20000 output

1 TDIV3$ logunif 50 20000 output

0 RESIZE1$ logunif 0.01 100 output

0 RESIZE2$ logunif 0.01 100 output

0 RESIZE3$ logunif 0.01 100 output

0 MUTATE unif 1e-7 1e-9 output

[RULES]

TDIV2$ > TDIV1$

TDIV3$ > TDIV2$

[COMPLEX PARAMETERS]

1 ANCSIZE1$ = NPOP3$ * RESIZE1$ output

1 ANCSIZE2$ = NPOP2$ * RESIZE2$ output

1 ANCSIZE3$ = NPOP2$ * RESIZE3$ output

Model_h.tpl

//Parameters for the coalescence simulation program:simcoal.exe

4

//Population effective sizes (number of genes)

NPOP0$

NPOP1$

NPOP2$

NPOP3$

//Samples sizes and samples time and inbreeding

28

50

18

28

//Growth rates: negative growth implies population expansion

0

0

0

0

//Number of migration matrices:

2

//migration matrix 0

0 0 0 0

0 0 0 0

0 0 0 0.005

0 0 0.005 0

//migration matrix 1

0 0 0 0

0 0 0 0

0 0 0 0

0 0 0 0

//historical event: time, source, sink, migrants, new deme size, growth rate, migr mat index

4 historical event

TDIV1$ 0 3 GF$ 1 0 0

TDIV1$ 0 2 1 RESIZE1$ 0 0

TDIV2$ 1 2 1 RESIZE2$ 0 1

TDIV3$ 3 2 1 RESIZE3$ 0 1

//Number of independent loci [chromosome]

1 0

//Per chromosome: Number of contiguous linkage Block: a block is a set of contiguous loci

1

//per Block:data type, number of loci, per gen recomb and mut rates

FREQ 1 0 MUTATE OUTEXP

Model_h.est

// Priors and rules file

// *********************

[PARAMETERS]

//#isInt? #name #dist.#min #max

//all N are in number of haploid individuals

1 NPOP0$ logunif 100 100000 output

1 NPOP1$ logunif 100 100000 output

1 NPOP2$ logunif 100 100000 output

1 NPOP3$ logunif 100 100000 output

1 TDIV1$ logunif 50 20000 output

1 TDIV2$ logunif 50 20000 output

1 TDIV3$ logunif 50 20000 output

0 GF$ unif 0 1 output

0 RESIZE1$ logunif 0.01 100 output

0 RESIZE2$ logunif 0.01 100 output

0 RESIZE3$ logunif 0.01 100 output

0 MUTATE unif 1e-7 1e-9 output

[RULES]

TDIV2$ > TDIV1$

TDIV3$ > TDIV2$

[COMPLEX PARAMETERS]

1 ANCSIZE1$ = NPOP2$ * RESIZE1$ output

1 ANCSIZE2$ = NPOP2$ * RESIZE2$ output

1 ANCSIZE3$ = NPOP2$ * RESIZE3$ output
